# Synergistic combination therapy delivered via layer‐by‐layer nanoparticles induces solid tumor regression of ovarian cancer

**DOI:** 10.1002/btm2.10429

**Published:** 2022-11-08

**Authors:** Stephanie Kong, Pearl Moharil, Abram Handly‐Santana, Natalie Boehnke, Richard Panayiotou, Victoria Gomerdinger, Gil Covarrubias, Ivan S. Pires, Ioannis Zervantonakis, Joan Brugge, Paula T. Hammond

**Affiliations:** ^1^ Koch Institute for Integrative Cancer Research, Massachusetts Institute of Technology Cambridge Massachusetts United States; ^2^ Department of Chemical Engineering Massachusetts Institute of Technology Cambridge Massachusetts United States; ^3^ Harvard Medical School Harvard University Boston Massachusetts United States; ^4^ Department of Bioengineering University of Pittsburgh Pittsburgh Pennsylvania United States

**Keywords:** cancer, combination therapy, drug delivery, layer‐by‐layer, nanoparticle, solid tumor, synergy

## Abstract

The majority of patients with high grade serous ovarian cancer (HGSOC) develop recurrent disease and chemotherapy resistance. To identify drug combinations that would be effective in treatment of chemotherapy resistant disease, we examined the efficacy of drug combinations that target the three antiapoptotic proteins most commonly expressed in HGSOC—BCL2, BCL‐XL, and MCL1. Co‐inhibition of BCL2 and BCL‐XL (ABT‐263) with inhibition of MCL1 (S63845) induces potent synergistic cytotoxicity in multiple HGSOC models. Since this drug combination is predicted to be toxic to patients due to the known clinical morbidities of each drug, we developed layer‐by‐layer nanoparticles (LbL NPs) that co‐encapsulate these inhibitors in order to target HGSOC tumor cells and reduce systemic toxicities. We show that the LbL NPs can be designed to have high association with specific ovarian tumor cell types targeted in these studies, thus enabling a more selective uptake when delivered via intraperitoneal injection. Treatment with these LbL NPs displayed better potency than free drugs in vitro and resulted in near‐complete elimination of solid tumor metastases of ovarian cancer xenografts. Thus, these results support the exploration of LbL NPs as a strategy to deliver potent drug combinations to recurrent HGSOC. While these findings are described for co‐encapsulation of a BCL2/XL and a MCL1 inhibitor, the modular nature of LbL assembly provides flexibility in the range of therapies that can be incorporated, making LbL NPs an adaptable vehicle for delivery of additional combinations of pathway inhibitors and other oncology drugs.

## 1 INTRODUCTION

Ovarian cancer remains the leading cause of death among gynecologic malignancies in the United States, with an overall 5‐year survival rate of 41%–49% that drops to 24%–30% for those with late stage diagnoses (Stage III/IV).^1^, ^2^ First line treatment of high grade serous ovarian cancer (HGSOC), the most common form of ovarian cancer, consists of surgical cytoreduction and platinum‐based chemotherapy. This often fails to eradicate residual disease, resulting in 70% patient relapse within 5 years.^3^ What drives relapse—particularly in patients with advanced‐stage solid ovarian cancers—is cancer cell resistance to nonspecific, cytotoxic platinum‐based treatment.^4^

In contrast to traditional chemotherapies, targeted therapies inhibit specific oncogenic pathways essential for tumor cell survival. For HGSOC, which lacks many identifiable therapeutically treatable point mutations, targeting the oncogenic and often‐dysregulated mitochondrial apoptotic pathway is especially attractive.^5^ BCL‐2 homology domain 3 (BH3) mimetics are drugs that mimic the inhibitory actions of proapoptotic BH3 only proteins by binding to antiapoptotic BCL‐2 family proteins (i.e., BCL2, BCL‐XL, MCL1), leading to reactivation of apoptotic machinery.^6^, ^7^ Inhibiting the action of antiapoptotic BCL‐2 family proteins has already shown promise in treating a number of human hematopoietic malignancies^8^ and non‐ovarian solid tumor xenografts.^6^, ^9^, ^10^ Although studies have reported synergistic interactions from co‐inhibition of BCL‐XL and MCL1 antiapoptotic proteins,^11^, ^12^, ^13^, ^14^, ^15^, ^16^, ^17^, ^18^, ^19^, ^20^ this inhibitor combination had yet to be investigated in treatment of solid tumors of HGSOC. Clinical evaluation of HGSOC tumors has correlated high expression of BCL‐XL/MCL1 with increased chemoresistance/recurrence. These two proteins were also found to be commonly expressed in HGSOC patient derived xenograft (PDX) models,^21^, ^22^ suggesting that targeted inhibition of BCL‐XL and MCL1 proteins is a promising strategy for overcoming mechanistic resistance.

Compared to other BH3 mimetics,^8^, ^23^ the translation of small molecule BCL‐XL and MCL1 targeted therapies is less clinically advanced due to toxicities and pharmacological challenges. While targeted inhibitors are selective for specific oncogenic pathways, their delivery to cells is nonspecific. Both BCL‐XL and MCL1 are present in healthy adult, non‐malignant tissue, and studies have reported thrombocytopenia^24^ and cardiac toxicities^25^, ^26^ for some BCL‐XL and MCL1 inhibitors. Additionally, many BCL‐XL and MCL1 inhibitors exhibit poor solubility and unfavorable pharmacokinetics, and thus represent suitable drug candidates that would benefit from the use of a delivery vehicle.^6^

Layer‐by‐layer nanoparticles (LbL NPs), formed from the electrostatic assembly of oppositely charged polyelectrolytes, have been shown to extend circulation time, limit off‐target toxicities, and enhance targeted therapeutic delivery to cancer cells.^27^, ^28^, ^29^, ^30^, ^31^ We have recently shown that LbL NPs designed with appropriate outer layer chemistries exhibit greater than 80% ovarian cancer tumor accumulation when delivered intraperitoneally and can be further functionalized with outer layer targeting chemistry that enhances penetration of solid tumors.^32^, ^33^, ^34^ Modular LbL architecture can be adapted for encapsulation of a wide range of therapeutics^27^, ^28^, ^29^, ^30^, ^31^, ^34^, ^35^ and cancer cell targeting chemistries,^32^, ^33^ and has scalable synthesis.^36^

We developed a synergistic LbL NP combination therapy for targeted delivery and treatment of HGSOC. We demonstrate that dual inhibition of BCL2/XL and MCL1 is synergistic in 4 out of 5 tested HGSOC models, and that treatment efficacy is significantly enhanced by LbL NP‐mediated delivery to cancer cells in vitro. In a metastatic mouse model of ovarian cancer, we observed that the LbL NP combination therapy eliminated nearly all metastatic solid tumor lesions at lower total drug dosages (6 mg/kg) than what has been previously reported in the literature for other mouse models.^10^, ^19^, ^37^ Additionally, we determined no overt toxicities from LbL NP combination treatment. Moreover, we demonstrate in vivo that it is the combination of BCL2/XL and MCL1 inhibition, rather than singular inhibition of either BCL2/XL or MCL1 that successfully induces regression of metastatic solid tumor lesions in HGSOC. We report here a synergistic combination therapy, delivered via LbL NPs, as a potential treatment for overcoming platinum resistance and inducing significant regression of solid tumors in HGSOC.

## 2 RESULTS AND DISCUSSION

### 2.1 BCL2/XL and MCL1 inhibitors act synergistically to treat HGSOC

To examine the effects of single and combination treatment with BCL2/XL and MCL1 inhibitors, we used ABT‐263 (a BCL2/XL inhibitor, BCL2/XLi) and S63845 (an MCL1 inhibitor, MCL1i). Navitoclax (ABT‐263) is under investigation in ongoing Phases 1, 2, and 3 studies. The drug is dosed orally, up to 300 mg in patients. The most common adverse event was reversible thrombocytopenia without clinically significant bleeding (88%). In preclinical mouse models, Navitoclax is most commonly administered orally at a dose of 100 mg/kg.^38^ S64315, the clinical‐trial version of S63845 (MIK665), is still in Phase 1 studies. It is under investigation in combination with other drugs for non‐ovarian cancer indications and is given intravenously.^39^ NCT03672695, a Phase 1 dose escalation study, investigates MIK665 in combination with venetoclax with dose ranging studies of MIK665 from 50–250 mg once weekly.

**FIGURE 1:**
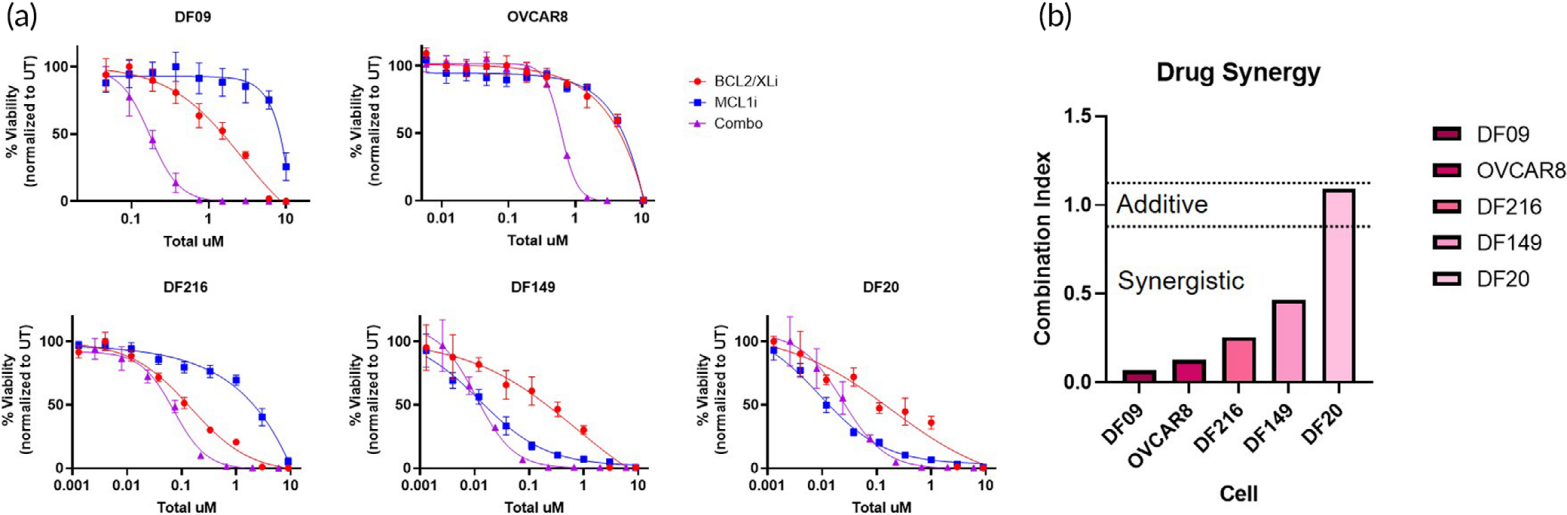
BCL2/XLi and MCL1i act synergistically to kill multiple cells of high grade serous ovarian cancer. (a) Dose–response curves of single‐drug and combination treatment of OVCAR8 and several patient derived xenograft (PDX) models. Percentage of viable cells following a 24 h incubation period is graphed against total drug concentration to ensure that apparent synergy is not a consequence of an overall increase in drug concentration. (b) Combination indices calculated for OVCAR8 and PDX DF09, DF216, and DF149 indicate the highly synergistic effect of combination treatment with BCL2/XLi (ABT‐263) and MCL1i (S63845). Combination treatment of DF20 appears to be additive

We carried out dose–response studies using ABT‐263 and S63845 in free drug form, on a panel of short term cultures of luciferase‐expressing PDXs obtained from chemoresistant HGSOCs^40^ (DF09, DF20, DF149, DF216) and the human ovarian cancer cell line OVCAR8. Viability was assessed via cell bioluminescence. Combined treatment with both inhibitors displayed greater efficacy than single drug treatment in all tested models except for DF20 (Figure 1a). Combination indices calculated from IC50 values show that combined BCL2/XL and MCL1 inhibition is synergistic in DF09, OVCAR8, DF216, and DF149 cells and additive in DF20 cells (Figure 1b). The strong synergy observed raised the possibility that combined inhibition of BCL2/XL and MCL1 could be effective for treatment of chemoresistant HGSOC. We examined this possibility further in PDX DF09 and OVCAR8 cells, for which strong synergy was observed with dual inhibition, despite modest single agent responses. We note that while responses to combined inhibition of both BCL2/XL and MCL1 were synergistic in most tested HGSOC cells, degree of synergy varied by PDX, and the effect was additive in DF20. Tumor cell heterogeneity is a potential cause of the observed differences in synergistic activity.^41^, ^42^ Previous work has shown that the relative abundance of antiapoptotic proteins varies between PDX models.^43^ Encapsulating inhibitors against BCL2, BCL‐XL, and MCL1 enables pathway inhibition across multiple models.

### 2.2 Loading and characterization of LbL NPs

**FIGURE 2:**
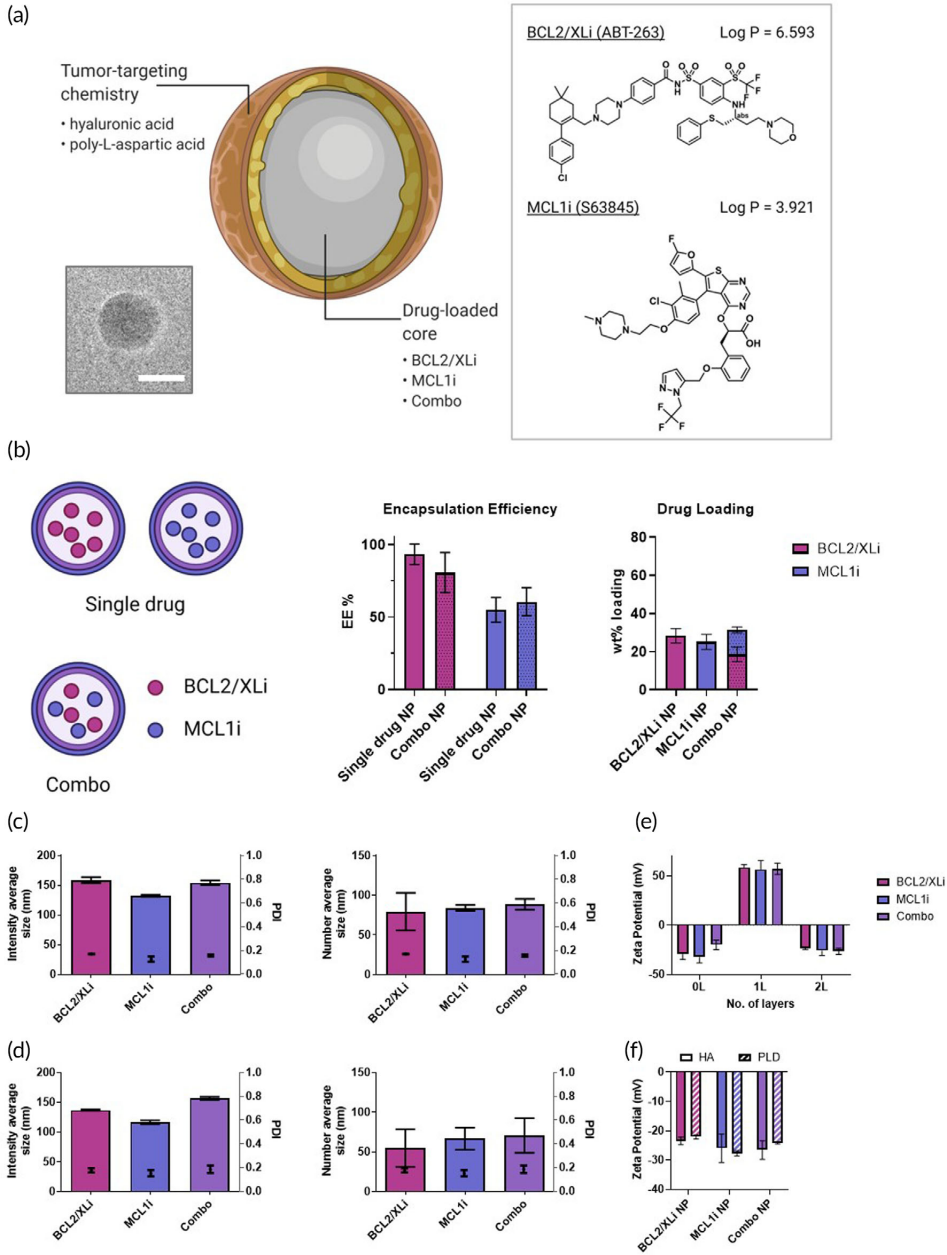
Characterization of drug‐loaded layer‐by‐layer nanoparticles (LbL NPs). (a) Drug‐loaded LbL NPs are formulated with an anionic poly‐lactic‐co‐glycolic acid core and layered with poly‐l‐arginine and hyaluronic acid (HA) or poly‐l‐aspartic acid (PLD) tumor‐targeting outer layer chemistry. Both BCL2/XLi and MCL1i are highly lipophilic and water‐insoluble. Cryogenic transmission electron microscopy Cryo‐TEM image is of a drug‐loaded LbL NP (scale bar = 50 nm). (b) LbL NPs are formulated as either single‐drug NPs (BCL2/XLi NP, MCL1i NP) or Combination NPs (Combo‐NP, BCL2/XLi + MCL1i). BCL2/XLi (Log *P* = 6.5) had higher encapsulation efficiency (EE) than MCL1i (Log *P* = 3.9) due its higher lipophilicity. Dotted bars denote BCL2/XLi or MCL1i drug loading for combination drug‐loaded NP. Total weight percent drug loading was 25%–31% for Single and Combo‐NPs. (c) HA‐layered NPs: Single‐drug BCL2/XLi NP (86 ± 15 nm), single‐drug MCL1i NP (85 ± 29 nm), and Combo‐NP (92 ± 19 nm) are similar in number average size. (d) PLD‐layered NPs are slightly smaller than HA‐NPs by number‐average size: Single‐drug Bcl‐2/xLi NP (55 ± 24 nm), Single‐drug MCL1i NP (67 ± 14 nm) and Combo‐NP (71 ± 22 nm). (e) Single‐drug and Combo‐NP final HA‐bilayer LbL NPs are similar in charge (BCL2/XLi NP – 23 ± 1, MCL1i NP – 26 ± 5, Combo‐NP – 28 ± 1). Charge switching confirms sequential layer‐by‐layer electrostatic assembly of oppositely charged polyelectrolytes 0L = unlayered; 1L = 1 layer; 2L = 2 layer. (f) HA and PLD‐layered NPs have similar final charge. Data are represented as the mean of three technical replicates. Standard deviation is represented by error bars.

LbL NPs were formulated with a drug‐loaded core and tumor‐targeting chemistry (Figure 2a) to improve the delivery of the lipophilic BCL2/XL and MCL1 inhibitors into cancer cells (Figure 2b). When used in free form with patients, these drugs are typically delivered systemically in oral formulations which cannot be targeted to the tumor. We elected to use poly‐lactic‐co‐glycolic acid (PLGA) to encapsulate the small molecule inhibitors for its biocompatibility, biodegradability, and anionic surface charge compatible with charge‐based LbL deposition. Drug cores were then layered with cationic poly‐l‐arginine (PLR), which has been previously used to promote endosomal escape of LbL NPs into the cytoplasm.^29^, ^35^, ^44^, ^45^ NPs were terminally layered with tumor‐targeting hyaluronic acid (HA) or poly‐l‐aspartic acid (PLD) outer layer chemistry. We selected HA because it is a known ligand of the CD44 receptor, overexpressed on HGSOC cells,^46^ and has been previously used by our lab and several others to target the CD44 receptor.^27^, ^28^, ^29^, ^30^, ^32^ We selected PLD based on our recent findings that LbL NPs coated with a surface layer of PLD have selective targeting interactions with a HGSOC model (OVCAR8).^32^ LbL NPs were formulated as single‐drug NPs (BCL2/XLi NP, MCL1i NP) and combination NPs (Combo‐NP, co‐encapsulation of BCL2/XLi and MCL1i) (Figure 2b). The method of nanoprecipitation^47^, ^48^ was utilized to load the inhibitors into the PLGA core. Tangential flow filtration^36^ was employed to remove unencapsulated drug prior to LbL deposition. High performance liquid chromatography (HPLC) was used to determine the encapsulation efficiency (EE) in the resulting LbL NPs (Figure 2b). We observed slightly higher EE for BCL2/XLi (Log *P* = 6.593) than MCL1i (Log *P* = 3.921). For Combo‐NPs, wt% loading of BCL2/xLi (18.6 ± 3.78) is slightly greater than wt% loading of MCL1i (12.8 ± 1.48). Accounting for differences in molecular weight, Combo‐NPs are loaded in approximately a 55:45 molar ratio of BCL2/xLi to MCL1i.

### 2.3 LbL NPs target HGSOC models in vivo

LbL NPs with different outer layers were assessed for in vivo colocalization with HGSOC models to select for optimal LbL NP targeting chemistry. OVCAR8‐Nude and DF09‐NSG models have significant tumor lesions in the omentum and along the fat covering the upper genital tract. Due to this disseminated tumor formation, colocalization of tumor bioluminescence signal and LbL NP fluorescence signal provides a stronger and more nuanced evidence of LbL NP homing to tumor cells. Colocalization analysis also allowed comparison of NP targeting across different tumor models more effectively; while the OVCAR8‐Nude model forms tumor nodules, the DF09‐NSG model forms a thin layer of tumor cells caking surfaces of the peritoneal space. Differences in tumor formation between models make NP accumulation in solid tumor impossible to compare across models using a “total tissue accumulation” approach.

**FIGURE 3:**
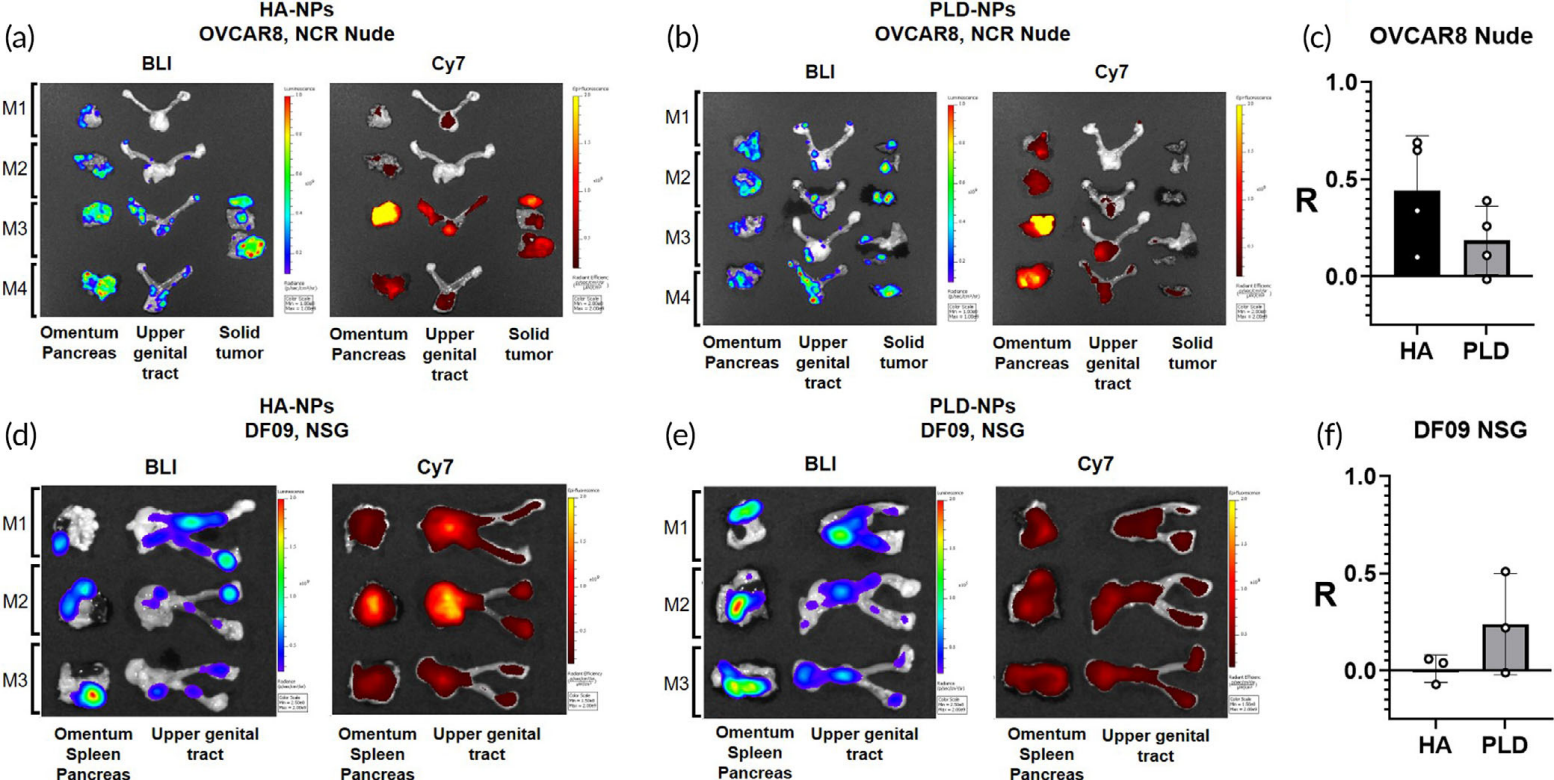
Layer‐by‐layer nanoparticles (LbL NPs) colocalize with OVCAR8 and DF09 tumors in vivo. LbL NP outer layer chemistry drives differences in degree of colocalization. Corresponding bioluminescence imaging (BLI) and Cy7‐LbL NPs for OVCAR8‐NCR Nude mice treated with (a) hyaluronic acid (HA)‐NPs (*N* = 4), (b) poly‐l‐aspartic acid (PLD)‐NPs (*N* = 4), (c) HA‐NPs preferentially target OVCAR8 cells in vivo. Pearson's correlation coefficient (*R*) for HA‐NPs (0.45 ± 0.14) is higher than PLD‐NPs (0.19 ± 0.09). Corresponding BLI and Cy7‐LbL NPs for DF09‐NSG mice treated with (d) HA‐NPs (*N* = 3) or (e) PLD‐NPs (*N* = 3) are shown. (f) PLD‐NPs preferentially target DF09 cells in vivo. Correlation is higher for PLD‐NPs (0.24 ± 0.15) is higher than HA‐NPs (0.01 ± 0.04). Values of *R* are given as mean ± standard error. ImageJ Coloc2 plugin was used to calculate correlation coefficients. To prevent bias from background signal and ensure that correlation coefficients are only being calculated from tissue signal, regions of interest (ROIs) of the organs were generated prior to running the analysis

Cy7‐labeled HA‐ and PLD‐coated LbL NPs (without inhibitors) were formulated to track NP localization in vivo and have similar size and charge to drug‐loaded HA‐ and PLD‐ coated LbL NPs (Figure S1). Tumor‐bearing mice were generated by intraperitoneal (IP) transplantation of luciferized OVCAR8, DF09, DF181, DF101^40^, ^49^ into either nude or NOD‐SCIDγ (NSG) mice and were allowed to engraft for 2 weeks (PDX models) or 4 weeks (OVCAR8 model) prior to NP treatment. A single dose of either HA‐NPs or PLD‐NPs was administered to the IP cavity and mice were sacrificed after 24 h. Organs containing solid tumor lesions (omentum/pancreas, upper genital tract [UGT]) were excised to evaluate tumor bioluminescence imaging (BLI) signal and NP (Cy7) signal. To determine the degree of tumor‐NP colocalization, a Pearson's *R* correlation coefficient was generated for the images using the ImageJ Coloc2 plugin. NP‐tumor cell colocalization was strongest for OVCAR8 and DF09 models (Figures 3 and S2). In the OVCAR8 Nude model, HA‐NPs (Figure 3a) had greater tumor cell colocalization than PLD‐NPs (Figure 3b). Pearson's *R* was higher for HA‐NPs (0.45 ± 0.14) than PLD‐NPs (0.19 ± 0.09) in OVCAR8 mice (Figure 3c). Conversely, in the DF09 NSG model, PLD‐NPs (*R* = 0.24 ± 0.15) had greater tumor cell colocalization than HA‐NPs (*R* = 0.01 ± 0.04) (Figure 3d,e). *R* values for DF181 and DF101 models indicated very weak correlation for PLD‐NPs (0.26 ± 0.32), and no correlation between HA‐NP and BLI signal in either model (DF181, *R* = 0.02 ± 0.1; DF101, *R* = ‐0.08 ± 0.04) (Figure S2). The differences in degree of NP‐tumor colocalization suggest that the optimal targeting chemistry of LbL NPs may depend on differences in cell surface protein expression among tumor models.^40^ The LbL architecture provides a tunable platform that enables altering of the outer layer targeting chemistry toward given tumor cell types, independent of drug composition. It is anticipated that different tumor cell surface markers or overexpressed proteins might be targeted with ligands or surface chemistries. The HA outer layer homes most efficiently to OVCAR8 due to its characteristic CD44 overexpression which is also found in some other high grade ovarian cancer models.^50^, ^51^ Our lab has demonstrated high affinity of the PLD outer layer for several ovarian cancer cell lines^32^; we have proposed that these less‐specific interactions may involve hydrogen bond interactions between PLD and glycans expressed on a number of HGSOC cancer cell types. In vivo imaging system (IVIS) images depicting relative levels of NP accumulation in tumor and healthy tissue show that NP accumulation was highest in tumors covering the omentum/UGT, and in the liver and kidney. NP accumulation was lowest in the heart, spleen, and lungs (Figure S3).

### 2.4 LbL NP‐mediated delivery improves in vitro efficacy of combination treatment

**FIGURE 4:**
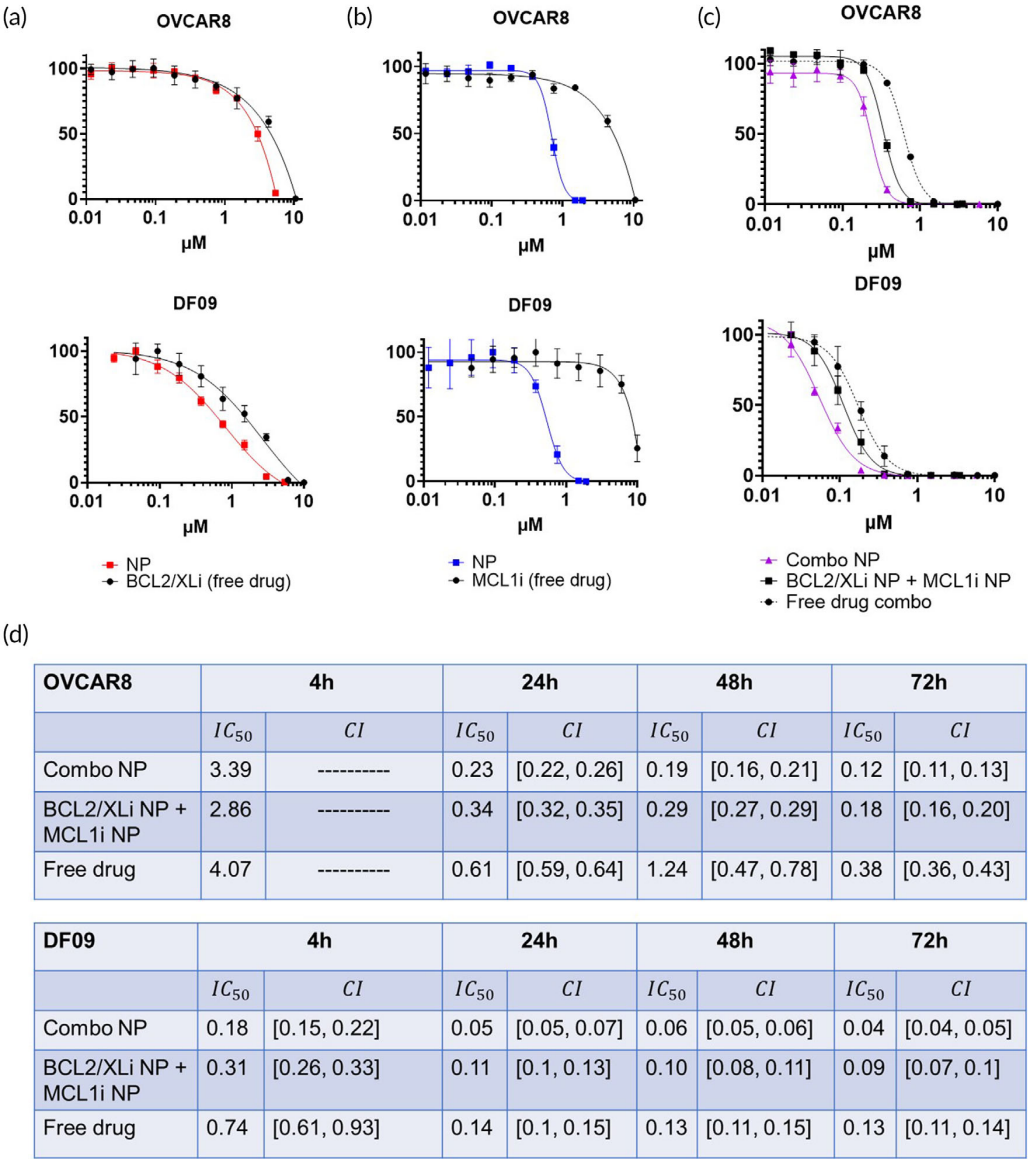
Layer‐by‐layer nanoparticles (LbL NPs) improve delivery of targeted inhibitors. Combo‐NPs improve synergistic killing of cancer cells over co‐treatment with single‐drug NPs. Cells were incubated with NP or free drug treatments for 4, 24, 48, and 72 h. Dose–response curves for (a) BCL2/XLi and (b) MCL1i demonstrate improved IC_50_ values for LbL NP treatment over free drug treatment. (c) Both Combo‐NP and co‐treatment of single‐drug NPs have lower IC_50_ than free drug treatment. Combo‐NP treatment had a lower IC_50_ than co‐treatment with single drug NPs. (d) IC_50_ values and 95% confidence intervals show that Combo‐NP treatment is most effective at 24 h, 48 h and 72 h for OVCAR8 and 4 h, 24 h, 48 h, 72 h for DF09. (a)–(c) display dose–response curves at 24 h. Percentage of cell viability signal following the incubation period is graphed against total drug concentration to ensure that apparent synergy is not a consequence of an overall increase in drug concentration. Note that at 4 h, the 95% confidence interval for OVCAR8 is indeterminable, meaning that the 95% confidence interval is too broad to accurately define the IC50

To assess the efficacy of LbL NP‐mediated delivery of BCL2/XLi and MCL1i, in vitro dose–response curves for drug‐loaded NPs were collected. For these studies we selected HGSOC cells with high in vivo NP‐tumor cell colocalization (OVCAR8, DF09). We focused our study on HA‐layered NPs over PLD‐layered NPs since HA‐NPs had the highest degree of colocalization in the OVCAR8 model, and unlike PLD, HA has well‐established targeting interactions between HA and CD44. Cancer cells were incubated for 4, 24, 48, and 72 h with NP or free‐drug treatments (Figures S4 and S5). Drug‐loaded HA‐LbL NPs have improved IC_50_ values over free drug treatments (Figure 4a,b), which we attribute to receptor‐mediated interactions between HA and CD44 leading to endocytosis.^32^ HA‐LbL NP encapsulation and delivery improve cytotoxic killing of BCL2/XLi (Figure 4a) and MCL1i (Figure 4b) in OVCAR8 and DF09 cells. PDX cells DF68 and DF149 show similar improvements in cytotoxicity with HA‐LbL NP‐mediated delivery (Figure S6). At 24, 48, and 72 h, Combo‐NP treatment had a lower IC_50_ than co‐treatment with single drug NPs (Figure 4c,d). Both NP treatments had lower IC_50_ than combination free drug. Based on the IC_50_ results, we advanced Combo‐NP as the optimal LbL NP for in vivo testing.

### 2.5 Combination NP therapy is safe and tolerable in Nude mice

**FIGURE 5:**
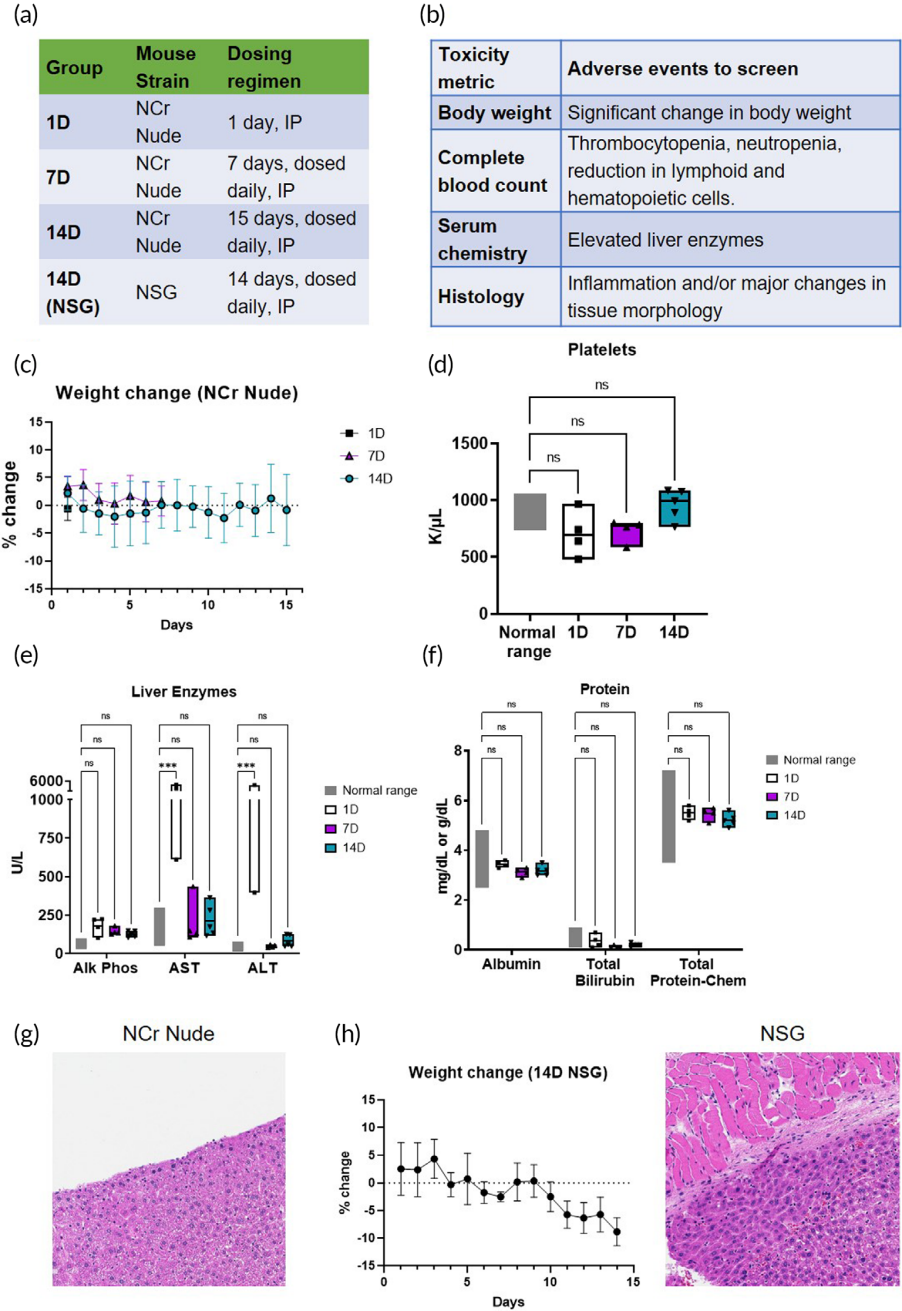
Toxicology assessment determines safety and tolerability of Combination nanoparticle (NP) therapy treatment in healthy NCr Nude and NSG mice. (a) Treatment groups designed to test compounding synergistic toxicities resulting from daily, continuous dosing; 1 day (1D) (*N* = 4), 7 days (7D) (*N* = 4), 14 days (14D) (*N* = 5) and 14D NSG (*N* = 5). (b) Mice were assessed with a full panel of toxicity metrics. (c) NCr Nude mice treated for 14D continuously had no significant weight loss and did not display signs of distress. (d) Platelet levels are normal, suggesting that layer‐by‐layer nanoparticle encapsulation of BCL2/XLi (ABT‐263) may mitigate dose‐limiting thrombocytopenia seen with free drug ABT‐263 administration. Gray bars indicate normal ranges for healthy mice, provided by Division of Comparative Medicine Comparative Pathology Lab. (e) Liver enzymes levels spiked for the 1D dosed cohort of Nude mice, but were within the normal range for 7D (continuous dosing) and 14D (continuous dosing) treatment groups. AST, Aspartate Aminotransferase; ALT, Alanine Transaminase (f) Despite the observed spike in liver enzyme levels seen with the 1D cohort, albumin, total bilirubin, and total protein levels remained within normal ranges for all treatment groups, suggesting minimal lasting toxicity from treatment. (g) Liver histology for 14D Nude mice appears normal. (h) There are strain dependent differences in treatment tolerability. 14D NSG mice did not tolerate the Combination NP treatment well, with a steady drop in weight over the 2 week dosing period (left). Liver histology indicates significant peritonitis and inflammation, with areas of fibrosis and collagen gluing the outer surface of the liver to the gut wall (right)

A number of significant toxicities have been reported with free drug administration of ABT‐263 (BCL2/XLi) and S63845 (MCL1i). ABT‐263 is known to cause BCL‐XL inhibition in platelets, resulting in significant, dose‐limiting, thrombocytopenia.^37^, ^52^ S63845 administration has been linked to a reduction in leukocytes and erythrocytes.^10^, ^19^ Additionally, combined BCL2/XL and MCL1 inhibition was previously reported to have synergistic toxicities impacting hepatic function.^17^, ^19^ Thus, we sought to assess the safety of Combo‐NP treatment in healthy Nude and NSG mice prior to efficacy studies in tumor‐bearing mouse models (OVCAR8 Nude, DF09 NSG). Healthy 6‐week old NCr Nude mice were treated with Combo‐NP at the maximum feasible dose (MFD) of 6 mg/kg/day. The MFD was determined based on maximal IP dosing volumes (200 μl) and the highest LbL NP concentration (~2 mg/ml) that would remain stable in solution. In order to evaluate if continuous treatment compounds toxicities, three treatment groups were established: (1) mice receiving a single one‐time dose (1D), (2) mice receiving daily doses for 7 days (7D), and (3) mice receiving daily doses for 14 days (14D) (Figure 5a). All doses were administered IP at the MFD.

A full panel of toxicological metrics was used to assess drug‐related toxicities (Figure 5b). Mice were monitored throughout treatment for signs of distress and significant change in body weight. Exactly 24 h after the last dose was administered, blood was collected and assayed for platelet, erythrocyte, and leukocyte health (via complete blood counts [CBCs]) and liver enzyme/protein levels via serum chemistry. Tissue sections were stained with hematoxylin and eosin to look for inflammation and major changes to tissue morphology. NCr Nude mice experienced no significant weight loss (Figure 5c). Platelet levels were within the normal ranges provided by MIT's Division of Comparative Medicine Comparative Pathology Lab (DCM CPL) (Figure 5d). While we observed that liver enzyme levels spiked in the 1D group after a single dose, these levels were within the normal range for 7D and 14D groups (Figure 5e). To assess hepatic function, protein levels were analyzed, and we found no lasting toxicity from continuous dosing treatment (Figure 5f). Lasting hepatic damage impacting liver function is expected to cause an increase in total bilirubin, and a decrease in albumin and total protein levels. All treatment groups were within normal ranges for total bilirubin, albumin, and protein levels. Tissue sections reveal no inflammation or major morphological changes to the liver (Figure 5g). Combo‐NP treatment did not cause damage to other major organs (kidney, spleen, heart, lungs) and levels of erythrocytes and leukocytes were within normal ranges (Figures S7 and S8).

Given that OVCAR8 and DF09 are established in different mouse strains (OVCAR8 NCr Nude, DF09 NSG), we sought to determine whether Combo‐NP treatment was equally tolerated in NSG mice. NSG mice were dosed under the 14D dosing schedule. Unlike NCr Nude mice, NSG mice did not tolerate this dosing scheme, suggesting strain‐dependent differences in tolerability to Combo‐NP treatment. We also observed that NSG mice continually lost weight throughout treatment (Figure 5g, left). Tissue sections reveal significant inflammation, fibrosis and collagen formation, along with adhesions of the gut wall to the surface of the liver, indicating peritonitis (Figure 5g, right). The strain dependent differences in treatment tolerability are likely a result of differences in immune competence among mouse strains; NSG mice have defective macrophages, resulting in slower drug clearance and a higher likelihood that overall drug concentration will compound through continuous daily dosing.^53^

### 2.6 Combination NP treatment causes regression of solid tumor burden

Due to the observed tolerability of healthy NCr Nude mice to Combo‐NP treatment (Figure 5), we selected the OVCAR8‐NCr Nude model for efficacy studies. This model establishes large IP tumors, and recapitulates the widely disseminated tumor growth and histology of HGSOC.^54^ Similar to advanced HGSOC found in patients, this tumor model forms nodal metastases covering the omentum, diaphragm, UGT, liver, and intestinal mesentery,^54^, ^55^ making it a suitable model for assessing the ability of Combo‐NP treatment to reduce solid tumor burden and metastatic lesions.

**FIGURE 6:**
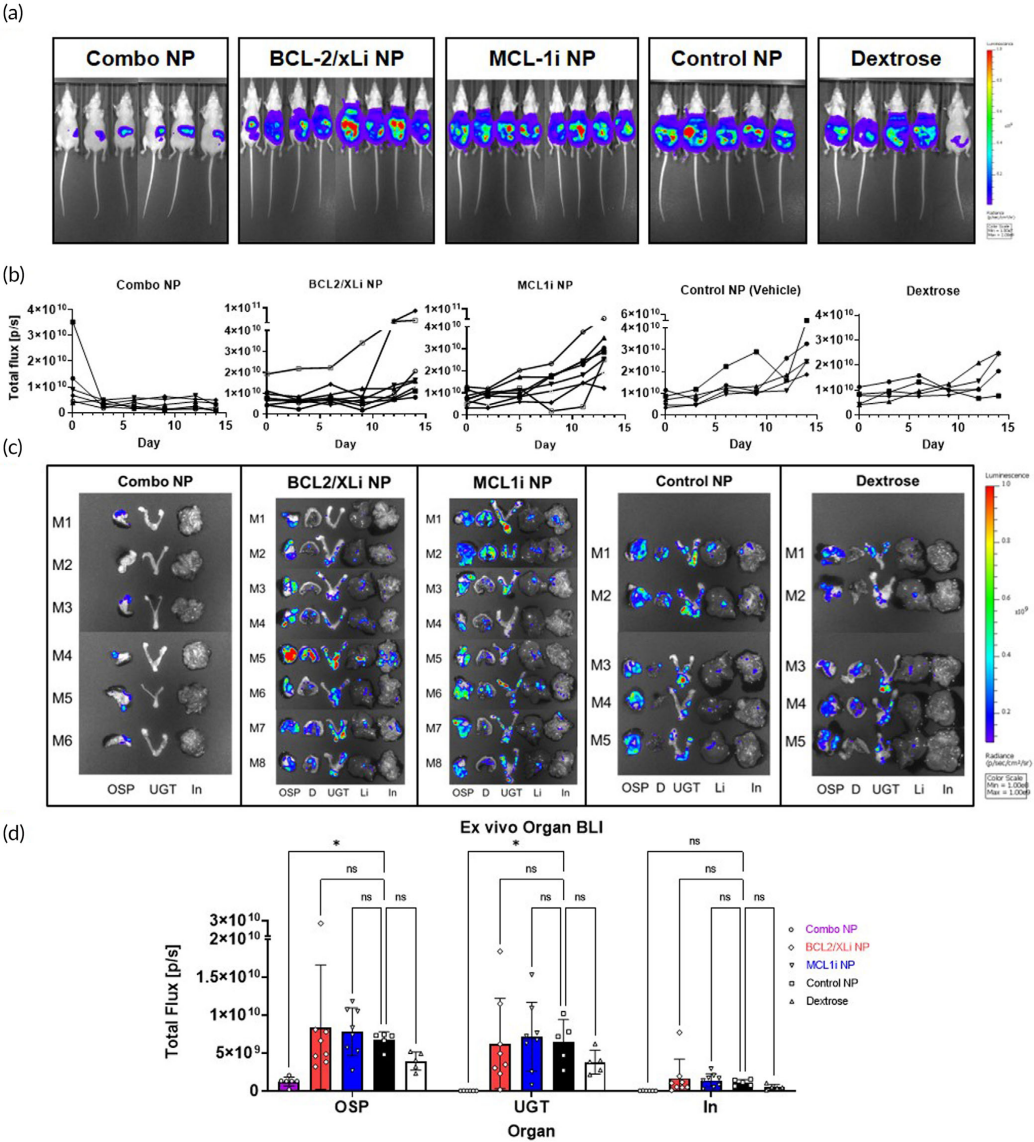
Combination layer‐by‐layer nanoparticle (LbL NP) therapy dramatically reduces tumor activity and solid tumor burden in high grade serous ovarian cancer (HGSOC). Treatment with BCL2/XLi NP or MCL1i NP does not significantly reduce tumor burden or metastasis in HGSOC. OVCAR8 NCr Nude mice with 4 week old metastatic tumors were treated with Combination LbL NP therapy for 14D continuously at the maximum feasible dose (MFD) of 6 mg/kg/day. Mice were treated daily at the MFD of 6 mg/kg/day for BCL2/XLi NP and 4 mg/kg/day for MCL1i NP. (a) Tumor burden drops dramatically in Combo‐NP treated mice (*N* = 6). Tumor burden continues to increase in all other groups: BCL2/XLi NP (*N* = 8), MCL1i NP (*N* = 8), Control‐NP (*N* = 5), and dextrose‐treated mice (*N* = 5). Tumor burden was tracked via bioluminescence imaging (BLI) signal with an in vivo imaging system (IVIS) throughout the course of treatment. Images are from Day 14 of treatment. Additional IVIS images provided in Figure S9A. (b) Combo‐NP treatment dramatically reduces tumor bioluminescence imaging (BLI) signal and suppresses tumor growth. BLI signal for individual mice is graphed for all treatment groups. (c) Combo‐NP treatment eliminates metastatic lesions on the upper genital tract, intestine, diaphragm, and liver. Organs containing visible solid tumor lesions were excised and imaged ex vivo for tumor activity (BLI signal). Combo‐NP treatment group did not have visible solid tumor lesions on the diaphragm, liver, upper genital tract, or intestine. (d) Differences in BLI signal are significant for the omentum/spleen/pancreas, and upper genital tract. D, diaphragm; In, intestine; Li, liver; OSP, omentum/spleen/pancreas; UGT, upper genital tract. *p < 0.05

Tumors were established via IP injection of OVCAR8 cells expressing luciferase in order to monitor tumor burden via bioluminescence imaging (BLI). HGSOC patients most often present at late stage. To more closely approximate this setting, we allowed our tumor models to establish significant solid tumor burden (average total flux 1 × 10^10^ p/s). Peritoneal tumors were allowed to grow for 4 weeks to ensure solid tumor formation prior to treatment. Prior to treatment, BLI values were used to sort mice and ensure equivalent tumor burden across all treatment groups. Mice that did not establish tumor (absence of tumor BLI signal) were excluded from the study. Mice were treated with Combo‐NP, Control NP (no drug), or a 5 wt% dextrose solution used to formulate NPs for isotonic injections. To assess in vivo synergy, we also evaluated the efficacy of single‐drug LbL NP treatment. Mice were dosed daily at the MFD (6 mg/kg/day) for 14D. BLI signal was assessed every 3 days throughout treatment. BLI imaging revealed a rapid reduction in tumor signal in response to Combo‐NP treatment (Figure 6a). This was in contrast to BCL2/XLi‐NP, MCL1i‐NP, Control‐NP, and Dextrose treatments which did not appear to impact tumor growth (Figure 6b). After 14D of continuous treatment, peritoneal organs with visible solid tumors were excised and imaged ex vivo for tumor BLI signal. Combo‐NP treatment significantly reduced solid tumor lesions on peritoneal organs, and nearly eliminated solid tumor nodal metastases covering the UGT, diaphragm, and liver (Figure 6c). The solid tumor lesions of mice receiving Combo‐NP are significantly smaller than those of Control‐NP treated mice (Figure 6d). BCL2/XLi‐NP and MCL1i‐NP treated mice did not experience a reduction in tumor burden (Figure 6a,b) or peritoneal metastases (Figure 6c,d). Differences in tumor BLI signal between Combo‐NP and both control groups (Control NP; Dextrose) were significant after just 6 days of treatment (Figure S9A,B). As expected there was no significant difference in BLI signal between Control‐NP and Dextrose treatment groups. As in healthy mice, all treatments were well‐tolerated in tumor‐bearing NCr Nude mice (Figure S9C). Weights of the Combo‐NP treated group remained constant throughout treatment.

### 2.7 Combination NP reduces the presence of nodal solid tumor lesions

**FIGURE 7:**
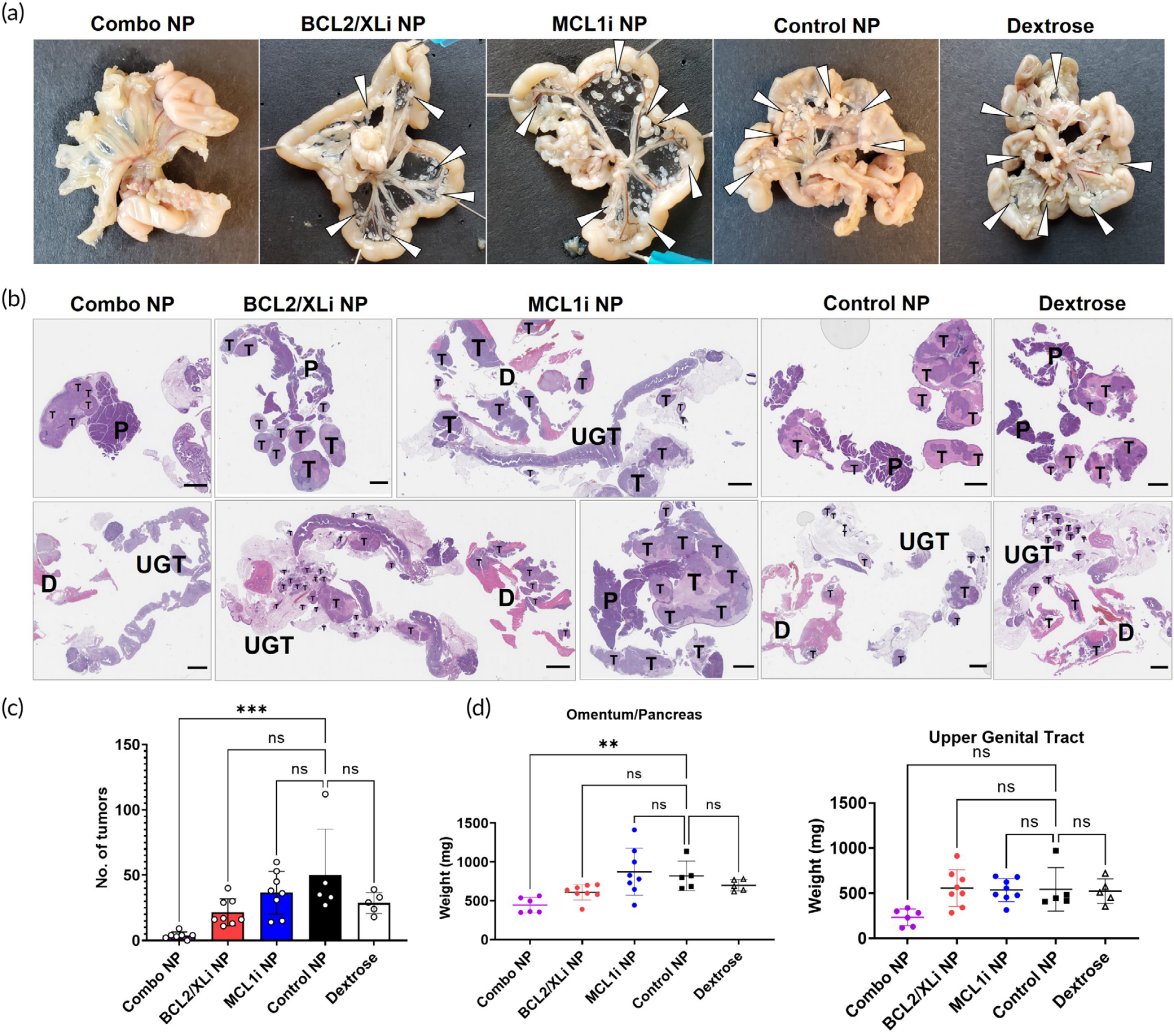
Combo‐nanoparticle (NP) treatment dramatically reduces the presence of nodal solid tumor lesions. (a) Although not visible with ex vivo in vivo imaging system imaging, photographs of the intestinal mesentery show significant differences in the presence of solid tumor. Solid tumor nodules (white pointers) that cover the mesentery of Control‐NP and Dextrose treated mice, are absent from Combo‐NP treated mice. (b) Representative tissue sections of the omentum (O), pancreas (P), upper genital tract (UGT), and diaphragm (D) are hematoxylin and eosin (H&E) stained and analyzed for the number of solid tumor implants. Solid tumor implants are labeled with a bold ‘T.’ As expected for the OVCAR8 model, the O/P region contains large solid tumor masses (top row). These tumor masses are significantly larger and necrotic in BCL2/XLi‐NP, MCL1i‐NP, Control‐NP, and Dextrose‐treated mice than in Combo‐NP treated mice. Small solid tumors cover the UGT and D of Control‐NP and Dextrose‐treated mice, but are absent in Combo‐NP treated mice. Scale bar is 2 mm. (c) Blinded quantification of solid tumor metastases coating peritoneal organs (omentum/pancreas, UGT/uterine horns, intestinal mesentery, D, liver). Combo‐NP treated mice have fewer tumors than Control‐NP and Dextrose‐treated mice. (d) Although not all solid tumor could be eliminated from the omentum/spleen/pancreas of Combo‐NP treated mice, this mass of solid tumor was greatly reduced. The weights of the O/spleen/P and UGT of Combo‐NP treated mice are much lower as a result of significantly reduced solid tumor burden coating these organs. Combo‐NP (*N* = 6), BCL2/XLi NP (*N* = 8), MCL1i NP (*N* = 8), Control‐NP (*N* = 5), and dextrose‐treatment (*N* = 5). ** *p* < 0.01, *** *p* < 0.001.

Extracted intestinal mesentery showed a dramatic reduction in nodal metastases with Combo‐NP treatment (Figure 7a). BCL2/XLi NP treatment did reduce solid tumor burden of the mesentery, but did not eliminate it. MCL1i NP treatment did not reduce solid tumor burden. To quantify total number of tumors, tissue sections of peritoneal organs containing solid tumor (omentum/pancreas, UGT/uterine horns, intestinal mesentery, diaphragm, liver) (Figure 7b), were analyzed by a pathologist blinded to experimental treatment groups. As expected for the OVCAR8 NCr Nude model, the omentum/pancreas (P) contained large solid tumor masses. Omental solid tumor masses were significantly larger in BCL2/XLi‐NP, MCL1i‐NP, Control‐NP and Dextrose‐treated mice. Small solid tumors covered the UGT and diaphragm (D) of BCL2/XLi‐NP, MCL1i‐NP, Control‐NP and Dextrose‐treated mice, but were absent in Combo‐NP mice. Thus, Combo‐NP treatment resulted in a significant reduction in the number of solid tumor implants. Solid tumor implants (T) are marked in representative tissue sections (Figure **7**b). Blinded quantification of solid tumor implants shows that total number of tumors was significantly lower for Combo‐NP treated mice (Figure 7c), and no significant difference in number of tumors between single‐drug NP treated mice and control groups (Control‐NP, Dextrose). The difference in solid tumor mass covering peritoneal organs was also reflected by ex vivo organ weights. Omentum/pancreas and UGT weights from Combo‐NP treated mice showed a significant reduction in mass (Figure 7d).

Reduced efficacy with single‐drug BCL2/XLi NP and MCL1i NP treatment show that only treatment with Combo‐NP reduces solid tumor, demonstrating the potent in vivo synergistic cytotoxicity of combined BCL2/XL and MCL1 inhibition. These results are in concordance with our earlier in vitro findings that HGSOC cells with a highly synergistic response to combination treatment saw minimal benefit from single‐drug treatment. This is also in agreement with a growing body of literature detailing the potent, cytotoxic effect of combination treatment with BCL‐XL and MCL1 inhibitors.^11^, ^12^, ^13^, ^14^, ^15^, ^16^, ^17^, ^18^, ^19^, ^20^ One mechanistic explanation for this effect could be that proapoptotic Bim proteins can shuttle between BCL‐XL and MCL1, and that single inhibition of one protein (BCL‐XL or MCL1) can lead to sequestration of Bim proteins by the other, thus limiting the amount of free proapoptotic proteins.^17^

All formulations tested in vivo on OVCAR8 NCr Nude tumor‐bearing mice were well tolerated; mice did not experience significant weight loss (Figure S9C). Levels of liver enzymes, protein levels, and blood urea nitrogen (BUN)/creatine were not statistically different among treatment groups suggesting normal liver and kidney function (Figure S10A,B). Mice did not experience a reduction in platelets with treatment (Figure S10C). And erthryocyte/leukocyte counts were not statistically different among treatments (Figure S11). Thus, all LbL NP treatments are well tolerated and demonstrably safe.

## 3 DISCUSSION

For HGSOC patients that relapse with advanced stage, chemotherapy‐resistant disease following first line treatment, few options exist. There is an immediate need for developing readily translatable, targeted therapies that downregulate oncogenic pathways in solid tumors of HGSOC. To address this, we developed a LbL NP combination therapy that utilizes tumor‐targeting chemistry to deliver a synergistic dose of BCL2/XLi (ABT‐263) and MCL1i (S63845) therapy to solid tumor cells. This represents a significant advancement for BCL‐XL and MCL1 inhibitors, which have faced challenges in clinical translation due to poor solubility and pharmacokinetics, as well as on‐target toxicities. To the best of our knowledge, this is the first report of a BCL2/XLi MCL1i synergistic therapy delivered via targeted NPs where significant regression of solid tumor metastasis in HGSOC was achieved. Given that residual disease remains the main prognostic factor of overall survival for HGSOC patients,^2^, ^3^ the ability of LbL NPs to target and treat microscopic solid tumor lesions represents a significant milestone for next‐stage therapeutics that combine targeted delivery with targeted therapies.

Significant tumor regression arising from synergistic inhibition of BCL‐XL and MCL1 has not yet been reported for HGSOC, but is known to induce solid tumor regression in other cancers.^11^, ^12^, ^13^, ^14^, ^15^, ^16^, ^17^, ^18^, ^19^, ^20^ We found that co‐inhibition of BCL2/XL and MCL1 was highly synergistic in a number of HGSOC models. Similar to a study by Bala et al., we found that NP‐mediated delivery of synergistic inhibitor therapies targeting the mitochondrial apoptosis pathway increases efficacy over free drug.^56^ One possible explanation for the lower IC_50_ of the Combo‐NP treatment compared to the IC_50_ of co‐treatment with BCL2/XLi NP + MCL1i NP is that Combo‐NP treatment ensures delivery of both inhibitors to a given cell. In extreme cases, when a subject receives co‐treatment with single‐drug LbL NPs, a small population of cells may receive only BCL2/XLi NPs and no MCL1i NPs, or only MCL1i NP and no BCL2/XLi NP. These cells will experience no synergistic cytotoxicity, and do not benefit from drug synergy. For synergistic inhibition, these effects may be more pronounced in vivo. Dose dilution decreases the likelihood that both free drugs of a synergistic combination will accumulate within tumor cells at a sufficient ratio to achieve maximal synergistic inhibition of oncogenic pathways. In the absence of NP encapsulation, free drugs with different chemistries will likely have different opsonization and off‐target accumulation, thus reducing the likelihood that both drugs will have the same degree of uptake by tumor cells. Co‐encapsulation within a tumor‐targeted NP may retain and deliver drugs at the appropriate ratio to its target cell. We note that co‐encapsulation can potentially standardize the pharmacokinetics of both drugs in vivo and reduce the need for complex dosing schemes typically required to ensure that each drug is present in sufficient quantities for simultaneous inhibition.

Our efficacy and toxicity studies in NCr Nude mice demonstrate a robust response and tolerance to LbL NP combination treatment. In order to replicate conditions seen with advanced stage ovarian cancer patients in our efficacy studies, we did not begin treatment until the cancer had metastasized throughout the peritoneal cavity and established significant solid tumor burden. Despite this, LbL NP combination therapy nearly eliminated all solid tumors from the peritoneal cavity, and significantly reduced tumor burden in the omentum. We report efficacy at a dose of 6 mg/kg/day—the lowest total dosage reported for inhibitor treatment with BCL2/XLi ABT‐263 and/or MCL1i S63845 to date.^10^, ^19^, ^37^ For patients, lowering the minimum efficacious dose through innovations in targeted delivery and targeted therapy has the additional benefits of reducing the cost of treatment and decreasing the risk of adverse effects from toxicity. Additionally, due to improved solubility, LbL NP therapy is suitable for IP administration, which has become the standard of care for treatment of peritoneal metastasis,^2^, ^57^, ^58^ enabling more localized delivery of BCL2/XL and MCL1 inhibitors.

Despite these advances, translation of LbL NP combination treatment will likely require enhanced tools for molecular profiling of key cancer cell targeting moieties and primed pathways of mechanistic resistance. We observed that tumor heterogeneity contributes to variations in NP‐localization and sensitivity to combination BCL2/XLi and MCL1i inhibition. To address these needs, studies by our lab are ongoing to better understand the molecular signatures that affect NP delivery to cancer cells^59^ and optimization for other tumor cells types. Future work will investigate the in vivo efficacy of combination therapies that target multiple oncogenic pathways at once.^43^, ^60^

## 4 CONCLUSION

This work seeks to establish a viable, potential therapy for ovarian cancer patients using drugs that would otherwise face severe dose‐limiting toxicities without LbL NP encapsulation and delivery. Inhibitors of antiapoptotic BCL2/XL and MCL1 proteins are receiving increasing attention for their abilities to target solid tumor and restore function to the commonly deregulated oncogenic mitochondrial apoptotic pathway. However, they face a number of challenges in their clinical translation. LbL NP encapsulation and targeted delivery can be used to safely and effectively deliver such synergistic combination therapies. We developed a LbL NP delivering a BCL2/XLi MCL1i synergistic combination therapy, and demonstrated its success in eliminating solid tumor peritoneal metastasis in a xenograft model of HGSOC. While future work remains to expand these studies to a wider array of HGSOC models, there is evidence in the literature indicating that a broad range of solid tumor carcinomas is sensitive to BCL2/XL and MCL1 targeted inhibition, and might similarly benefit from combining targeted treatment with targeted delivery using LbL NPs. We also note that the modular architecture of LbL NPs has been used to incorporate a wide range of therapeutic modalities^27^, ^28^, ^29^, ^30^, ^31^, ^34^, ^35^ and targeting moieties^32^, ^33^ by interchanging core/inner layer and outer layer chemistries, respectively. While this work demonstrated efficacious delivery of BCL2/XLi and MCL1i, LbL architecture may be adapted for encapsulation of different drug combinations (i.e., by using a different core) as well as cell surface markers (i.e., by layering a different anionic polymeric targeting chemistry), and therefore has the potential to enable targeted delivery of other small molecule targeted therapies.

## 5 MATERIALS AND METHODS

5.1

#### 5.1.1 Drug‐loaded Lbl NP synthesis

Drug‐loaded polymeric LbL NP cores were formed via nanoprecipitation described previously.^61^ PLGA 50:50 (7000–17,000 kDa, Sigma‐Aldrich) was co‐dissolved with pan‐BCL2/XL inhibitor ABT‐263 and MCL1 inhibitor S63845 (Selleck Chemicals) into a 1 ml solution of acetone at a 4:1:1 PLGA: BCL2/XLi: MCL1i weight ratio. A 28G syringe was used to inject the 1 ml polymer‐drug solution into 10 ml spinning MilliQ water. Acetone was allowed to evaporate from the solution for 24 h to form drug‐loaded cores.

Unencapsulated drug was removed via tangential flow filtration as described previously^36^ with 3–5 buffer exchanges into fresh MilliQ water. Drug‐loaded cores were subsequently layered with PLR (38.5 kDa; Alamanda Polymers) at a 1:0.25 PLGA:PLR weight ratio. Excess, unlayered polyelectrolyte was removed with tangential flow filtration after five buffer exchanges into MilliQ water. HA‐LbL NPs were formed by adding HA (20 kDa; LifeCore Biomedical) as the second layer, at a 1:0.5 core:HA weight ratio. PLD‐LbL NPs were formed by instead adding PLD (14 kDa, Alamanda Polymers) as the second layer, at a 1:0.25 core:PLD weight ratio. All polymers were used without modifications. The ratio of polyelectrolytes was previously optimized using a process outlined by Decher et al. for enabling stable charge conversion and particle stability.^62^ Thus, polyelectrolyte layering ratios are chosen in order to achieve colloidal stability (*ζ* ± 30 mV) and ensure sufficiently similar anionic charge for both PLD‐coated and HA‐coated NPs (Figure 2e). Final LbL NP drug content was quantified via HPLC using the absorbance signals at 280 nm (for ABT‐263) and fluorescence signal at 312/410 (for S63845).

#### 5.1.2 HPLC for quantification of drug‐loading

ABT263 and S63845 characterization was carried out on an Agilent 1200 system with G1322A degasser; G1311A quaternary Pump; G1367B HiP‐ALS (autosampler); G1321A FLD; G1315D DAD. Stationary phase and gradient profiles are noted below.

**Table:** 

*t* (min)	% Acetonitrile +0.05% TFA	% Water + 0.05% TFA
0	5	95
1	15	85
8	75	25
10	99	1
12	99	1

Drugs were dissolved in dimethyl sulfoxide (DMSO) and a SunFire C18 Column (100 Å, 5 μm, 4.6 mm × 150 mm) was used with a gradient of 5%–99% acetonitrile + 0.05% trifluoroacetic acid (TFA)/H_2_O + 0.05% TFA over 12 min with a flow rate of 1.4 ml/min.

#### 5.1.3 Transmission electron microscopy

Images of drug‐loaded LbL NPs were acquired using a JEOL 2100 FEG TEM. Samples were prepared in deionized (DI) water and subsequently frozen for imaging by the Koch Institute Nanotechnology Materials Facility. LbL NP solution was deposited on a lacey copper grid coated with a continuous carbon film and blotted to remove excess sample without damaging the carbon layer by Gatan Cryo Plunge III. The grid was mounted on a Gatan 626 single tilt cryo‐holder equipped in the TEM column. The specimen and holder tip were cooled by liquid‐nitrogen. Imaging on the JEOL 2100 FEG microscope was done using minimum dose method to avoid sample damage under the electron beam. The microscope was operated at 200 kV and with a magnification of 10,000–60,000 for assessing particle size and distribution. All images were recorded on a Gatan 2kx2k UltraScan CCD camera.

#### 5.1.4 Cy7‐labeled LbL NP synthesis

Cy7‐labeled LbL NPs for in vivo biodistribution studies were similarly formed via nanoprecipitation. Cyanine7 dicarboxylic acid (Cy7, Lumiprobe) in DMSO was co‐dissolved with PLGA into 300 μl of acetone at a 100:0.25 PLGA: Cy7 weight ratio. A 28G syringe was used to inject the polymer‐drug solution into 10 ml spinning MilliQ water. Acetone was allowed to evaporate from the solution for 24 h to form Cy7‐labeled cores.

Unconjugated Cy7 dye was removed via tangential flow filtration with 15–20 buffer exchanges into MilliQ water. Complete removal of excess dye was confirmed by separation of dye‐labeled cores and buffer solution using ultracentrifugation tubes (Pall Nanosep Centrifugal Devices with Omega Membrane, 30K), and subsequent quantification of free dye fluorescence in the Nanosep filtrate at Ex/Em 745/800 (emission bandwidth 30 nm) with a Tecan Pro Plate reader. As with drug‐loaded LbL NP cores, dye‐labeled LbL cores were layered at a 1:0.25 PLGA:PLR weight ratio, followed by tangential flow filtration to remove excess polyelectrolyte. Using these PLR‐layered cores, HA‐LbL NPs were then formed via a 1:0.5 core:HA layering weight ratio. PLD‐LbL NPs were formed via a 1:0.25 core:PLD layering weight ratio.

#### 5.1.5 Tissue culture

Both OVCAR8 cells and PDX cells were engineered to express luciferase as described previously.^40^, ^49^ OVCAR8 cells were cultured in RPMI 1640 (Corning) supplemented with 10% fetal bovine serum (Gibco) and 1% penicillin streptomycin (Corning). OVCAR8 cells were not used past Passage 20. PDX cells were cultured in MCDB106/M 199 media + 2% fetal bovine serum (FBS) +1% Pen/Strep^43^ and not used beyond Passage 8. PDXs were derived from ascites fluid of HGSOC chemo‐naïve patients (DF09, DF20) or patients that have been treated with multiple chemotherapies (DF101, DF149, DF216).

#### 5.1.6 Luciferase viability assay

Luciferized OVCAR8 or PDX cells were seeded at 10,000 cells/well into poly‐l‐lysine coated 96‐well plates with the appropriate cell culture media to a volume of 90 μl/well. OVCAR8 cells were given 24 h to adhere at 37°C. After 24 h, 10 μl LbL NPs in water or free drug dissolved in DMSO and water was added to each well. After the incubation period (4, 24, 48, or 72 h), 100 μl of 250 μg/ml d‐luciferin (PerkinElmer) was added to each well and bioluminescence signal was read after a predetermined incubation period (10‐min incubation for OVCAR8 cells, 5‐min incubation for PDX cells). Incubation periods were determined a priori to optimize the assay for maximal signal and minimal signal change with time. Bioluminescence signal was normalized to untreated control wells on each plate. Untreated control wells were included on each plate to mitigate the effects of bioluminescence signal drop off with time. Normalized viability data were fit to an inhibitor–response curve (variable slope, four parameters) with GraphPad Prism. Absolute IC_50_ values were calculated from interpolation of normalized data. Data are graphed as percentage of viable cells normalized to untreated wells versus total drug concentration in the well (if more than a single drug is present). Combination index was calculated from IC_50_ values of single drug and combination drug using the Chou–Talalay Method^63^:

(1) $$CI_{\mathrm{IC}50}=\frac{{\left[A\right]}_{\mathrm{AB}}}{{\left[A\right]}_{A}}+\frac{{\left[B\right]}_{\mathrm{AB}}}{{\left[B\right]}_{B}}.$$

Where A and B represent Drugs A and B, respectively, and AB represents the combination of Drugs A and B. [A]_AB_ represents the IC_50_ concentration of Drug A for the combination treatment AB.

#### 5.1.7 Animal studies

All animal experiments were approved by the Massachusetts Institute of Technology Committee on Animal Care (CAC) under protocol 0818‐062‐21 and were conducted under the oversight of the DCM. All reporting of animal work is compliant with Animal Research: Reporting of In Vivo Experiments (ARRIVE) guidelines. Mice were housed in groups of 4–5 mice/cage within Association for Asessment and Accreditation of Laboratory Animal Care (AAALAC) accredited animal facilities maintained by MIT DCM. All mice were acclimated to the animal facility for a minimum of 5 days upon arrival and all cages were housed in the same room. Treatments were administered in no particular order. For all analysis, a single animal is considered a single experimental unit. Mice were weighed daily and monitored for signs of distress. Mice were observed daily, with the intention to euthanize if severe weight loss (>20%), loss of consciousness, or impending morbidity were observed. All animals were euthanized at end of study by CO_2_ asphyxiation, followed by cervical dislocation. For humane reasons, death was not used as an endpoint.

#### 5.1.8 HGSOC orthotopic mouse models

OVCAR8‐GFP/Luc mouse models were established by inoculating 6 week old female NCr Nude (sp/sp, Taconic) or NSG (NOD‐*scid* IL2Rg^null^, Jackson) mice via IP injection of 2 million cells/mouse. OVCAR8‐GFP/Luc cells were allowed to grow in culture for 1 week prior to tumor inoculation. PDX models were established by inoculating 6 week old female NSG mice via IP injection. PDX cells defrosted from frozen storage and reconstituted in WI‐TOC media prior to injections. For all models, tumor burden was assessed via weekly BLI using an IVIS. Mice were treated with 200 μL of 15 mg/ml d‐luciferin (Perkin Elmer) in Dulbecco's phosphate‐buffered saline (DPBS) and imaged for BLI signal with IVIS after 15 min.

#### 5.1.9 In vivo NP biodistribution

OVCAR8 and PDX mouse models were treated IP with fluorescently tagged LbL NPs, 4 weeks and 2 weeks post‐tumor inoculation, respectively. Each mouse model was treated with either (1) Cy7‐labeled HA LbL NPs (OVCAR8‐NCR Nude *N* = 4, DF09‐NSG *N* = 3) or (2) Cy7‐labeled PLD LbL NPs (OVCAR8‐NCR Nude *N* = 4, DF09‐NSG *N* = 3) to assess the relative biodistribution of HA and PLD‐coated LbL NPs among different HGSOC models. After 24 h, mice were sacrificed, and organs were imaged ex vivo for Cy7 NP signal and tumor cell BLI signal. To determine colocalization of NP and tumor signal, excised organs were incubated in 1 mg/ml d‐luciferin in DPBS for 2 min, followed by BLI imaging and fluorescence imaging on IVIS. Correlation coefficients were generated from tumor BLI and Cy7 NP fluorescence images with ImageJ's coloc2 plugin. Photographs of the organs generated during the IVIS imaging process were used to generate ROIs, to eliminate biasing of the correlation coefficient from background signal.

#### 5.1.10 Toxicology studies in healthy mice

Healthy 6 week old NCr Nude and NSG mice were treated via IP injection BCL2/XLi and MCL1i combination drug‐loaded HA‐LbL NPs (Combo‐NP) at a total drug dosage of 6 mg/kg/day (MFD). In the final step of LbL NP formulation, NPs must be filtered and concentrated via tangential flow filtration, which subjects the NPs to shear forces. The MFD was determined by the maximum feasible concentration at which LbL NPs remain stable in solution. Empirically, we have observed that HA‐coated LbL NPs cannot be concentrated above 2 mg/ml (PLGA content) without seeing evidence of aggregation (by dynamic light scattering) post‐concentration. Treatment cohorts included NCr Nude mice receiving (1) a single dose (*N* = 4), (2) 7D continuous dosing (*N* = 4), and (3) 14D continuous dosing (*N* = 5), and NSG mice receiving 14D continuous dosing (*N* = 5). As PDX models are established in NSG mice, an NSG mouse cohort treated under the most rigorous dosing schedule (14D, continuous) was included to test for strain‐dependent differences in toxicity. Mice were weighed daily and monitored for signs of distress. Mice were sacrificed 24 h after the last dose and blood was collected via cardiac puncture for CBC and serum chemistry analysis. Whole blood for CBC analysis was collected in K3 ethylenediaminetetraacetic acid (EDTA) tubes (Sarstedt). Whole blood for serum chemistry analysis was collected in 1.1 ml Serum Separator tubes (Sarstedt) containing a serum gel with clotting activator. Blood samples were submitted to MIT DCM CPL for same‐day processing. Excised organs were prepared as described under Section 5.1.12 and stained tissue sections stained with hematoxylin/eosin were evaluated for major changes in tissue morphology and/or inflammation.

#### 5.1.11 Efficacy and safety in HGSOC mouse model

Orthotopic OVCAR8‐GFP/Luc models were established in 6 week old female NCr Nude mice as described under Section 5.1.8. Tumors were allowed to grow for 4 weeks prior to treatment, and tumor burden was monitored weekly via IVIS BLI imaging. The day before treatment began, BLI burden was re‐measured and mice were organized to have equal tumor burden across treatment groups. Mice that did not successfully establish tumors were excluded from the experiment. Treatment cohorts were as follows: Combo‐NP (*N* = 6), BCL2/XLi NP (*N* = 8), MCL1i NP (*N* = 8), Control‐NP (*N* = 5), and dextrose‐treated mice (*N* = 5). A total of 32 mice were treated. All treatment groups were dosed IP at 6 mg/kg/day continuously for 14D. Mice were weighed daily and monitored for signs of distress. BLI signal was assessed every 3 days from the start of dosing to monitor tumor burden throughout treatment. Mice were sacrificed 24 h after the last dose. Blood samples for CBC and serum chemistry were collected via cardiac puncture, and submitted to MIT DCM CPL for same‐day processing. Excised organs were imaged ex vivo for BLI signal coming from microscopic metastatic lesions, following the d‐luciferin procedure described in section: In vivo NP biodistribution. Organs were weighed and prepared for fixation.

#### 5.1.12 Histopathology

For toxicology studies, excised organs (liver, kidney, spleen, heart, lungs) were fixed for 24 h with 4% methanol‐free formaldehyde solution. After 24 h, organs were transferred to cassettes and remained in 70% ethanol prior to paraffin embedding and sectioning by the Koch Institute Histology Core Facility. Sections were stained with hematoxylin/eosin and digitally scanned at 20× with an Aperio slide scanner. Sections were analyzed for inflammation and changes in tissue morphology by a board certified pathologist blinded to treatment group.

For efficacy studies, excised organs (liver, kidney, spleen, heart, lungs, UGT, mesentery, diaphragm, omentum/pancreas) were fixed for 24 h with 4% methanol‐free formaldehyde solution. After 24 h, organs were transferred to cassettes and remained in 70% ethanol prior to paraffin embedding and sectioning by the Koch Institute Histology Core Facility. Tumor‐containing tissue sections were stained with hematoxylin/eosin and digitally scanned at 20× with an Aperio slide scanner. Sections were analyzed for total number of solid tumor implants by a board certified pathologist blinded to treatment group.

#### 5.1.13 Statistical analysis

Statistics were calculated using GraphPad Prism. Data are reported as mean ± standard deviation unless otherwise specified. A two‐tailed Student's *t*‐test was used for comparisons between two experimental groups. Multiple comparisons were conducted using 2way‐ANOVA with post hoc Bonferroni analysis. *p* Values below 0.05 were considered significant. Sample sizes for efficacy experiments were calculated a priori using G*Power and a point‐biserial correlation with a very large effect size (*d* = 0.90), significance level (*α* = 0.05, two‐tailed, by convention), and level of power (1 − *β* = 0.80, by convention). All subjects across all experimental groups were included in analysis.

## AUTHOR CONTRIBUTIONS

**Stephanie Kong:** Conceptualization (lead); formal analysis (lead); investigation (lead); methodology (equal); project administration (lead); resources (equal); visualization (lead); writing – original draft (lead); writing – review and editing (lead). **Pearl Moharil:** Conceptualization (equal); formal analysis (equal); investigation (equal); project administration (equal); resources (equal). **Abram Handly‐Santana:** Conceptualization (supporting); formal analysis (supporting); investigation (supporting); methodology (equal); resources (equal); writing – review and editing (supporting). **Natalie Boehnke:** Conceptualization (equal); funding acquisition (equal); investigation (supporting); methodology (equal); project administration (supporting); resources (equal); writing – review and editing (supporting). **Richard Panayiotou:** Conceptualization (supporting); formal analysis (supporting); investigation (supporting); methodology (equal); resources (equal); writing – review and editing (supporting). **Victoria Gomerdinger:** Investigation (supporting); resources (equal); writing – review and editing (supporting). **Gil Covarrubias:** Investigation (supporting); resources (supporting); writing – review and editing (supporting). **Ivan Pires:** Investigation (supporting); resources (supporting); writing – review and editing (supporting). **Ioannis Zervantonakis:** Funding acquisition (equal). **Joan Brugge:** Conceptualization (lead); funding acquisition (lead); project administration (equal); resources (equal); supervision (lead); writing – review and editing (lead). **Paula T. Hammond:** Conceptualization (lead); funding acquisition (lead); project administration (lead); resources (lead); writing – review and editing (lead).

## Supporting information


**Figure S1.** Fluorescent Cy7 layer‐by‐layer nanoparticles (LbL NPs) used for in vivo biodistribution studies have similar size and charge to drug‐loaded BCL2/XLi, MCL1i NPs and Combo‐NPs. All LbL NPs are formulated with an anionic poly‐lactic‐co‐glycolic acid core and layered with poly‐l‐arginine and hyaluronic acid (HA) or poly‐l‐aspartic acid (PLD) tumor‐targeting outer layer chemistry. HA and PLD‐layered NPs have similar final charge. 0L = unlayered; 1L = 1 layer; 2L = 2 layer.
**Figure S2.** In vivo Nanoparticle (NP)‐tumor cell colocalization varies with layer‐by‐layer nanoparticle (LbL NP) outer layer chemistry and high grade serous ovarian cancer model. OVCAR8 and DF09 models have the highest degree of colocalization with LbL NPs. OVCAR8 cells display a high degree of colocalization with hyaluronic acid (HA)‐NPs in both Nude and NSG mouse strains. Patient derived xenograft tumor models established in NSG mice colocalize with poly‐l‐aspartic acid‐LbL NPs only. Table lists Pearson's *R* correlation coefficient (mean ± standard error) for each tumor model.
**Figure S3.** In vivo imaging system images show relative levels of nanoparticle (NP) accumulation in the heart, lungs, liver spleen, kidney, and tumor tissue. Layer‐by‐layer nanoparticle signal is normalized by tissue weight. For both (A) OVCAR8‐Nude and (B) DF09‐NSG models dosed with either hyaluronic acid (HA) or poly‐l‐aspartic acid (PLD)‐coated NPs, percent total radiant efficiency per gram (% TRE/g) signal was highest in the tumors covering the omentum/upper genital tract, liver and kidney of most models. % TRE/g signal was lowest in the heart, spleen, and lungs. H = heart; K = kidney; Li = liver; Lu = lung; O = omentum; P = pancreas; S = spleen; T = tumor; UGT = upper genital tract.
**Figure S4.** OVCAR8 cells are treated with drug‐loaded hyaluronic acid‐layer‐by‐layer nanoparticles and free drug for 4, 24, 48, and 72 h incubation periods. (A) For all time points, nanoparticle (NP)‐mediated delivery improved IC50 over the free drug treatment. (B) For 24, 48, and 72 h incubation periods, Combo‐NP treatment has the highest efficacy—higher than co‐treatment with single‐drug NPs. Both combination NP treatments and, in some cases, single‐drug MCL1i NP treatment had higher efficacy than combination free drug treatment.
**Figure S5.** DF09 cells are treated with drug‐loaded layer‐by‐layer nanoparticles and free drug for 4, 24, 48, and 72 h incubation periods. (A) For all time points, nanoparticle (NP)‐mediated delivery improved IC50 over the free drug treatment. (B) For 24, 48, and 72 h incubation periods, Combo‐NP treatment has the highest efficacy—higher than co‐treatment with single‐drug NPs (BCL2/XLi NP + MCL1i NP), or single‐drug NP treatment (BCL2/XLi NP, MCL1i NP).
**Figure S6.** Layer‐by‐layer nanoparticle encapsulation and delivery improve therapeutic efficacy of BCL2/XLi, MCL1i, and combination drug treatment for DF68 and DF149. (A) BCL2/XLi nanoparticle (NP) has improved IC50 over free drug BCL2/XLi for DF68 and DF149. (B) MCL1i NP has improved IC50 over free drug MCL1i for DF68 and DF149. (C) Combo‐NP has improved IC50 over free drug combination treatment for DF68 and DF149.
**Figure S7.** Blood counts are within normal ranges for 1day, 7 days and 14 days Nude mice under daily dosing with Combo‐NP treatment. (A) Leukocyte levels are within normal ranges. (B) Red blood cell levels and morphology are normal.
**Figure S8.** Histology images show no observable toxicity to heart, lung, liver, spleen, and kidney tissue of NCr Nude mice exposed to the Combo‐NP. Tissues were excised from mice bearing OVCAR8‐tumors, stained with hematoxylin and eosin, and reviewed by an expert pathologist. NSG mice do not tolerate the Combo‐NP drug as well as the NCr Nude mice. NSG mouse liver tissue sections reveal significant inflammation, fibrosis and collagen formation, along peritonitis.
**Figure S9.** Treatment with Combo‐NP significantly reduces tumor burden. Treatment with BCL2/XLi NP or MCL1i NP does not significantly reduce tumor burden. Tumor burden (bioluminescence imaging [BLI] signal) was measured throughout treatment course using Iin vivo imaging system. (A) Tumor burden decreases with treatment of Combo‐NP. (B) Tumor burden grows despite treatment with BCL2/XLi NP or MCL1i NP. (C) Tumor BLI signal of the Combo‐NP group compared to both control groups (Control‐NP, Dextrose) is significant as early as 6 days after treatment initiation (Combo‐ vs. Control‐NP, *p* = 0.048; Combo‐NP vs. Dextrose, *p* = 0.007). (D) All treatments are well tolerated. Mice experience no significant change in body weight.
**Figure S10.** Liver and kidney function are normal for all treatment groups. Platelet levels and platelet morphology were normal for all groups. (A) Levels of liver enzymes and levels of albumin, bilirubin, and total protein (indicating liver function) are normal. (B) All treatment groups had normal kidney function, as indicated by BUN and creatine levels. (C) Platelet levels and platelet morphology were normal for all groups.
**Figure S11.** Blood counts are normal for all treatment groups. No significant difference in (A) red blood cell health (B,C) leukocyte health were detected among treatment groups.

## Data Availability

The data that support the findings of this study are available from the corresponding author upon reasonable request.

## References

[1] Siegel RL , Miller KD , Fuchs HE , Jemal A . Cancer Statistics, 2021. CA Cancer J Clin. 2021;71(1):7‐33. 33433946 10.3322/caac.21654

[2] Kurnit KC , Fleming GF , Lengyel E . Updates and new options in advanced epithelial ovarian cancer treatment. Obstet Gynecol. 2021;137(1):108‐121. 33278287 10.1097/AOG.0000000000004173 PMC7737875

[3] du Bois A , Reuss A , Pujade‐Lauraine E , Harter P , Ray‐Coquard I , Pfisterer J . Role of surgical outcome as prognostic factor in advanced epithelial ovarian cancer: a combined exploratory analysis of 3 prospectively randomized phase 3 multicenter trials. Cancer. 2009;115(6):1234‐1244. 19189349 10.1002/cncr.24149

[4] Bivona TG , Doebele RC . A framework for understanding and targeting residual disease in oncogene‐driven solid cancers. Nat Med. 2016;22(5):472‐478. 27149220 10.1038/nm.4091 PMC5384713

[5] Lopez J , Tait SWG . Mitochondrial apoptosis: killing cancer using the enemy within. Br J Cancer. 2015;112(6):957‐962. 25742467 10.1038/bjc.2015.85 PMC4366906

[6] Kehr S , Vogler M . It's time to die: BH3 mimetics in solid tumors. Biochim Biophys Acta ‐ Mol Cell Res. 2021;1868(5):118987. 33600840 10.1016/j.bbamcr.2021.118987

[7] Wood KC . Overcoming MCL‐1‐driven adaptive resistance to targeted therapies. Nat Commun. 2020;11(1):531. 31988312 10.1038/s41467-020-14392-z PMC6985132

[8] Roberts AW , Davids MS , Pagel JM , et al. Targeting BCL2 with Venetoclax in relapsed chronic lymphocytic leukemia. New Engl J Med. 2015;374(4):311‐322. 26639348 10.1056/NEJMoa1513257 PMC7107002

[9] Oltersdorf T , Elmore SW , Shoemaker AR , et al. An inhibitor of Bcl‐2 family proteins induces regression of solid tumours. Nature. 2005;435(7042):677‐681. 15902208 10.1038/nature03579

[10] Kotschy A , Szlavik Z , Murray J , et al. The MCL1 inhibitor S63845 is tolerable and effective in diverse cancer models. Nature. 2016;538(7626):477‐482. 27760111 10.1038/nature19830

[11] Lee EF , Harris TJ , Tran S , et al. BCL‐XL and MCL‐1 are the key BCL‐2 family proteins in melanoma cell survival. Cell Death Dis. 2019;10(5):342. 31019203 10.1038/s41419-019-1568-3 PMC6482196

[12] Kehr S , Haydn T , Bierbrauer A , Irmer B , Vogler M , Fulda S . Targeting BCL‐2 proteins in pediatric cancer: dual inhibition of BCL‐XL and MCL‐1 leads to rapid induction of intrinsic apoptosis. Cancer Lett. 2020;482:19‐32. 32145345 10.1016/j.canlet.2020.02.041

[13] Carter RJ , Milani M , Butterworth M , et al. Exploring the potential of BH3 mimetic therapy in squamous cell carcinoma of the head and neck. Cell Death Dis. 2019;10(12):912. 31801952 10.1038/s41419-019-2150-8 PMC6892862

[14] Abdul Rahman SF , Muniandy K , Soo YK , et al. Co‐inhibition of BCL‐XL and MCL‐1 with selective BCL‐2 family inhibitors enhances cytotoxicity of cervical cancer cell lines. Biochem Biophys Rep. 2020;22:100756. 32346617 10.1016/j.bbrep.2020.100756 PMC7183162

[15] Greaves G , Milani M , Butterworth M , et al. BH3‐only proteins are dispensable for apoptosis induced by pharmacological inhibition of both MCL‐1 and BCL‐X(L). Cell Death Differ. 2019;26(6):1037‐1047. 30185825 10.1038/s41418-018-0183-7 PMC6748112

[16] Cho SY , Han JY , Na D , et al. A novel combination treatment targeting BCL‐X(L) and MCL1 for KRAS/BRAF‐mutated and BCL2L1‐amplified colorectal cancers. Mol Cancer Ther. 2017;16(10):2178‐2190. 28611106 10.1158/1535-7163.MCT-16-0735

[17] Weeden CE , Ah‐Cann C , Holik AZ , et al. Dual inhibition of BCL‐XL and MCL‐1 is required to induce tumour regression in lung squamous cell carcinomas sensitive to FGFR inhibition. Oncogene. 2018;37(32):4475‐4488. 29743589 10.1038/s41388-018-0268-2

[18] Goodwin CM , Rossanese OW , Olejniczak ET , Fesik SW . Myeloid cell leukemia‐1 is an important apoptotic survival factor in triple‐negative breast cancer. Cell Death Differen. 2015;22(12):2098‐2106. 10.1038/cdd.2015.73 PMC4816117 26045046

[19] Mukherjee N , Skees J , Todd KJ , et al. MCL1 inhibitors S63845/MIK665 plus Navitoclax synergistically kill difficult‐to‐treat melanoma cells. Cell Death Dis. 2020;11(6):443. 32513939 10.1038/s41419-020-2646-2 PMC7280535

[20] Soderquist RS , Crawford L , Liu E , et al. Systematic mapping of BCL‐2 gene dependencies in cancer reveals molecular determinants of BH3 mimetic sensitivity. Nat Commun. 2018;9(1):3513. 30158527 10.1038/s41467-018-05815-z PMC6115427

[21] Williams J , Lucas PC , Griffith KA , et al. Expression of Bcl‐xL in ovarian carcinoma is associated with chemoresistance and recurrent disease. Gynecol Oncol. 2005;96(2):287‐295. 15661210 10.1016/j.ygyno.2004.10.026

[22] Shigemasa K , Katoh O , Shiroyama Y , et al. Increased MCL–1 expression is associated with poor prognosis in ovarian carcinomas. Jpn J Cancer Res. 2002;93(5):542‐550. 12036450 10.1111/j.1349-7006.2002.tb01289.x PMC5927039

[23] Souers AJ , Leverson JD , Boghaert ER , et al. ABT‐199, a potent and selective BCL‐2 inhibitor, achieves antitumor activity while sparing platelets. Nat Med. 2013;19(2):202‐208. 23291630 10.1038/nm.3048

[24] Gandhi L , Camidge DR , De Oliveira MR , et al. Phase I study of navitoclax (ABT‐263), a novel bcl‐2 family inhibitor, in patients with small‐cell lung cancer and other solid tumors. J Clin Oncol. 2011;29(7):909‐916. 21282543 10.1200/JCO.2010.31.6208 PMC4668282

[25] FDA Places Trials of MCL‐1 Inhibitor on Clinical Hold. *ASH Clinical News*; 2019.

[26] Bolomsky A , Vogler M , Köse MC , et al. MCL‐1 inhibitors, fast‐lane development of a new class of anti‐cancer agents. J Hematol Oncol. 2020;13(1):173. 33308268 10.1186/s13045-020-01007-9 PMC7731749

[27] Gu L , Deng ZJ , Roy S , Hammond PT . A combination RNAi‐chemotherapy layer‐by‐layer nanoparticle for systemic targeting of KRAS/P53 with cisplatin to treat non–small cell lung cancer. Clin Cancer Res. 2017;23(23):7312‐7323. 28912139 10.1158/1078-0432.CCR-16-2186 PMC5712246

[28] Dreaden EC , Morton SW , Shopsowitz KE , et al. Bimodal tumor‐targeting from microenvironment responsive hyaluronan layer‐by‐layer (LbL) nanoparticles. ACS Nano. 2014;8(8):8374‐8382. 25100313 10.1021/nn502861t PMC4148172

[29] Deng ZJ , Morton SW , Ben‐Akiva E , Dreaden EC , Shopsowitz KE , Hammond PT . Layer‐by‐layer nanoparticles for systemic codelivery of an anticancer drug and siRNA for potential triple‐negative breast cancer treatment. ACS Nano. 2013;7(11):9571‐9584. 24144228 10.1021/nn4047925 PMC3870477

[30] Dreaden EC , Kong YW , Morton SW , et al. Tumor‐targeted synergistic blockade of MAPK and PI3K from a layer‐by‐layer nanoparticle. Clin Cancer Res. 2015;21(19):4410‐4419. 26034127 10.1158/1078-0432.CCR-15-0013 PMC4624301

[31] Barberio AE , Smith SG , Correa S , et al. Cancer cell coating nanoparticles for optimal tumor‐specific cytokine delivery. ACS Nano. 2020;14:11238‐11253. 32692155 10.1021/acsnano.0c03109 PMC7530125

[32] Correa S , Boehnke N , Barberio AE , et al. Tuning nanoparticle interactions with ovarian cancer through layer‐by‐layer modification of surface chemistry. ACS Nano. 2020;14:2224‐2237. 31971772 10.1021/acsnano.9b09213 PMC7062411

[33] Boehnke N , Dolph KJ , Juarez VM , Lanoha JM , Hammond PT . Electrostatic conjugation of nanoparticle surfaces with functional peptide motifs. Bioconjug Chem. 2020;31(9):2211‐2219. 32786506 10.1021/acs.bioconjchem.0c00384 PMC7895459

[34] Boehnke N , Correa S , Hao L , et al. Theranostic layer‐by‐layer nanoparticles for simultaneous tumor detection and gene silencing. Angew Chem Int Ed. 2020;59(7):2776‐2783. 10.1002/anie.201911762 PMC7002217 31747099

[35] Correa S , Boehnke N , Deiss‐Yehiely E , Hammond PT . Solution conditions tune and optimize loading of therapeutic polyelectrolytes into layer‐by‐layer functionalized liposomes. ACS Nano. 2019;13(5):5623‐5634. 30986034 10.1021/acsnano.9b00792 PMC6980385

[36] Correa S , Choi KY , Dreaden EC , et al. Highly scalable, closed‐loop synthesis of drug‐loaded, layer‐by‐layer nanoparticles. Adv Funct Mater. 2016;26(7):991‐1003. 27134622 10.1002/adfm.201504385 PMC4847955

[37] Wilson WH , O'Connor OA , Czuczman MS , et al. Navitoclax, a targeted high‐affinity inhibitor of BCL‐2, in lymphoid malignancies: a phase 1 dose‐escalation study of safety, pharmacokinetics, pharmacodynamics, and antitumour activity. Lancet Oncol. 2010;11(12):1149‐1159. 21094089 10.1016/S1470-2045(10)70261-8 PMC3025495

[38] Mohamad Anuar NN , Nor Hisam NS , Liew SL , Ugusman A . Clinical review: Navitoclax as a pro‐apoptotic and anti‐fibrotic agent. Front Pharmacol. 2020;11:564108. 33381025 10.3389/fphar.2020.564108 PMC7768911

[39] Clinical Trials Using Mcl‐1 Inhibitor MIK665. https://www.cancer.gov/about-cancer/treatment/clinical-trials/intervention/mcl-1-inhibitor-mik665. Accessed July 11, 2022.

[40] Liu JF , Palakurthi S , Zeng Q , et al. Establishment of patient‐derived tumor xenograft models of epithelial ovarian cancer for preclinical evaluation of novel therapeutics. Clin Cancer Res. 2017;23(5):1263‐1273. 27573169 10.1158/1078-0432.CCR-16-1237 PMC5332350

[41] Stewart JM , Shaw PA , Gedye C , Bernardini MQ , Neel BG , Ailles LE . Phenotypic heterogeneity and instability of human ovarian tumor‐initiating cells. Proc Natl Acad Sci USA. 2011;108(16):6468‐6473. 21451132 10.1073/pnas.1005529108 PMC3081039

[42] Dobbin ZC , Katre AA , Steg AD , et al. Using heterogeneity of the patient‐derived xenograft model to identify the chemoresistant population in ovarian cancer. Oncotarget. 2014;5(18):8750‐8764. 25209969 10.18632/oncotarget.2373 PMC4226719

[43] Zervantonakis IK , Iavarone C , Chen H‐Y , et al. Systems analysis of apoptotic priming in ovarian cancer identifies vulnerabilities and predictors of drug response. Nat Commun. 2017;8(1):365. 28848242 10.1038/s41467-017-00263-7 PMC5573720

[44] Choi KY , Correa S , Min J , et al. Binary targeting of siRNA to hematologic cancer cells In vivo using layer‐by‐layer nanoparticles. Adv Funct Mater. 2019;29(20):1900018. 31839764 10.1002/adfm.201900018 PMC6910249

[45] Deng ZJ , Morton SW , Bonner DK , Gu L , Ow H , Hammond PT . A plug‐and‐play ratiometric pH‐sensing nanoprobe for high‐throughput investigation of endosomal escape. Biomaterials. 2015;51:250‐256. 25771015 10.1016/j.biomaterials.2015.02.013 PMC4387846

[46] Lesley J , Hyman R , English N , Catterall JB , Turner GA . CD44 in inflammation and metastasis. Glycoconj J. 1997;14(5):611‐622. 9298694 10.1023/a:1018540610858

[47] Fessi H , Puisieux F , Devissaguet JP , Ammoury N , Benita S . Nanocapsule formation by interfacial polymer deposition following solvent displacement. Int J Pharm. 1989;55(1):R1‐R4.

[48] Barichello JM , Morishita M , Takayama K , Nagai T . Encapsulation of hydrophilic and lipophilic drugs in PLGA nanoparticles by the nanoprecipitation method. Drug Dev Ind Pharm. 1999;25(4):471‐476. 10194602 10.1081/ddc-100102197

[49] Dreaden EC , Kong YW , Quadir MA , et al. RNA‐peptide nanoplexes drug DNA damage pathways in high‐grade serous ovarian tumors. Bioeng Transl Med. 2018;3(1):26‐36. 29376131 10.1002/btm2.10086 PMC5773954

[50] Martincuks A , Li P‐C , Zhao Q , et al. CD44 in ovarian cancer progression and therapy resistance‐a critical role for STAT3. Front Oncol. 2020;10:589601. 33335857 10.3389/fonc.2020.589601 PMC7736609

[51] The Human Protein Atlas . [Webpage]. 21.1:https://www.proteinatlas.org/. Accessed June 25, 2022.

[52] Tolcher AW , LoRusso P , Arzt J , et al. Safety, efficacy, and pharmacokinetics of navitoclax (ABT‐263) in combination with erlotinib in patients with advanced solid tumors. Cancer Chemother Pharmacol. 2015;76(5):1025‐1032. 26420235 10.1007/s00280-015-2883-8

[53] Shultz LD , Schweitzer PA , Christianson SW , et al. Multiple defects in innate and adaptive immunologic function in NOD/LtSz‐scid mice. J Immunol. 1995;154(1):180‐191. 7995938

[54] Mitra AK , Davis DA , Tomar S , et al. In vivo tumor growth of high‐grade serous ovarian cancer cell lines. Gynecol Oncol. 2015;138(2):372‐377. 26050922 10.1016/j.ygyno.2015.05.040 PMC4528621

[55] Matulonis UA , Sood AK , Fallowfield L , Howitt BE , Sehouli J , Karlan BY . Ovarian cancer. Nat Rev Dis Primers. 2016;2(1):16061. 27558151 10.1038/nrdp.2016.61 PMC7290868

[56] Bala Tannan N , Manzari MT , Herviou L , et al. Tumor‐targeted nanoparticles improve the therapeutic index of BCL2 and MCL1 dual inhibition. Blood. 2021;137(15):2057‐2069. 33067607 10.1182/blood.2020008017 PMC8057264

[57] Coccolini F , Fugazzola P , Montori G , Ansaloni L , Chiarugi M . Intraperitoneal chemotherapy for ovarian cancer with peritoneal metastases, systematic review of the literature and focused personal experience. J Gastrointest Oncol. 2021;12(Suppl 1):S144‐S181. 33968435 10.21037/jgo-2020-06 PMC8100719

[58] Armstrong DK , Bundy B , Wenzel L , et al. Intraperitoneal cisplatin and paclitaxel in ovarian cancer. N Engl J Med. 2006;354(1):34‐43. 16394300 10.1056/NEJMoa052985

[59] Boehnke N , Straehla JP , Safford HC , et al. Massively parallel pooled screening reveals genomic determinants of nanoparticle delivery. Science. 2022;377(6604):eabm5551. 35862544 10.1126/science.abm5551 PMC10249039

[60] Iavarone C , Zervantonakis IK , Selfors LM , et al. Combined MEK and BCL‐2/XL inhibition is effective in high‐grade serous ovarian cancer patient–derived xenograft models and BIM levels are predictive of responsiveness. Mol Cancer Ther. 2019;18(3):642‐655. 30679390 10.1158/1535-7163.MCT-18-0413 PMC6399746

[61] Vrancken MN , Claeys DA . Process for Encapsulating Water and Compounds in Aqueous Phase by Evaporation; U.S. Patent No. 3,523,906. 11 Aug 1970.

[62] Schneider G , Decher G . Functional core/shell nanoparticles via layer‐by‐layer assembly. Investigation of the experimental parameters for controlling particle aggregation and for enhancing dispersion stability. Langmuir. 2008;24(5):1778‐1789. 18225923 10.1021/la7021837

[63] Chou T‐C . Drug combination studies and their synergy quantification using the Chou‐Talalay method. Cancer Res. 2010;70(2):440‐446. 20068163 10.1158/0008-5472.CAN-09-1947

