# Supplementary material for: Synergistic combination therapy delivered via layer‐by‐layer nanoparticles induces solid tumor regression of ovarian cancer
Source: Bioeng Transl Med. 2022 Nov 8;8(2):e10429. doi: 10.1002/btm2.10429 (PMC10013771; doi:10.1002/btm2.10429)
Supplement: Supplementary file 1 — Figure S1. Fluorescent Cy7 layer‐by‐layer nanoparticles (LbL NPs) used for in vivo biodistribution studies have similar size and charge to drug‐loaded BCL2/XLi, MCL1i NPs and Combo‐NPs. All LbL NPs are formulated with an anionic poly‐lactic‐co‐glycolic acid core and layered with poly‐l‐arginine and hyaluronic acid (HA) or poly‐l‐aspartic acid (PLD) tumor‐targeting outer layer chemistry. HA and PLD‐layered NPs have similar final charge. 0L = unlayered; 1L = 1 layer; 2L = 2 layer. Figure S2. In vivo Nanoparticle (NP)‐tumor cell colocalization varies with layer‐by‐layer nanoparticle (LbL NP) outer layer chemistry and high grade serous ovarian cancer model. OVCAR8 and DF09 models have the highest degree of colocalization with LbL NPs. OVCAR8 cells display a high degree of colocalization with hyaluronic acid (HA)‐NPs in both Nude and NSG mouse strains. Patient derived xenograft tumor models established in NSG mice colocalize with poly‐l‐aspartic acid‐LbL NPs only. Table lists Pearson's R correlation coefficient (mean ± standard error) for each tumor model. Figure S3. In vivo imaging system images show relative levels of nanoparticle (NP) accumulation in the heart, lungs, liver spleen, kidney, and tumor tissue. Layer‐by‐layer nanoparticle signal is normalized by tissue weight. For both (A) OVCAR8‐Nude and (B) DF09‐NSG models dosed with either hyaluronic acid (HA) or poly‐l‐aspartic acid (PLD)‐coated NPs, percent total radiant efficiency per gram (% TRE/g) signal was highest in the tumors covering the omentum/upper genital tract, liver and kidney of most models. % TRE/g signal was lowest in the heart, spleen, and lungs. H = heart; K = kidney; Li = liver; Lu = lung; O = omentum; P = pancreas; S = spleen; T = tumor; UGT = upper genital tract. Figure S4. OVCAR8 cells are treated with drug‐loaded hyaluronic acid‐layer‐by‐layer nanoparticles and free drug for 4, 24, 48, and 72 h incubation periods. (A) For all time points, nanoparticle (NP)‐mediated delivery improved IC50 over [file BTM2-8-e10429-s001.docx]

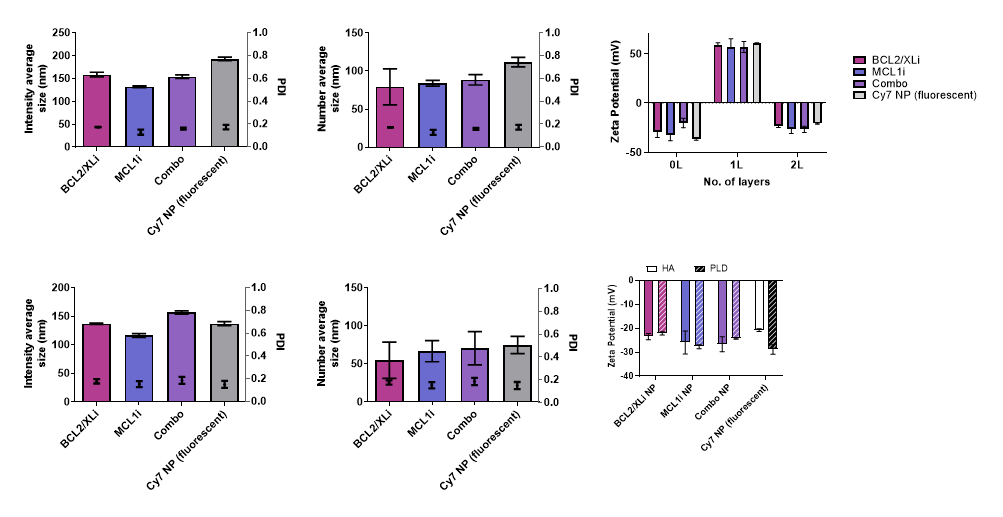


**Figure S1. Fluorescent Cy7 LbL NPs used for in vivo biodistribution studies have similar size and charge to drug-loaded BCL2/XLi, MCL1i NPs and Combo-NPs.** All LbL NPs are formulated with an anionic PLGA core and layered with poly-L-arginine (PLR) and hyaluronic acid (HA) or poly-L-aspartic acid (PLD) tumor-targeting outer layer chemistry. 0L = unlayered; 1L = 1 layer; 2L = 2 layer. HA and PLD-layered NPs have similar final charge.


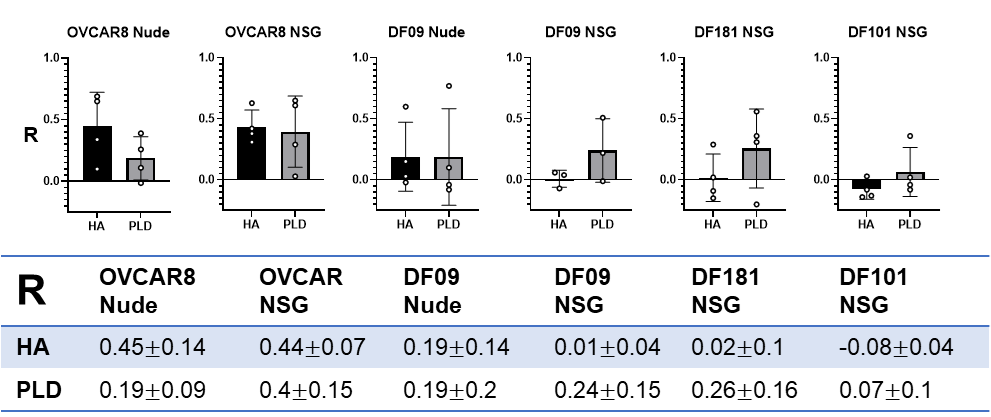


**Figure S2. In vivo NP-tumor cell colocalization varies with LbL NP outer layer chemistry and HGSOC model.** OVCAR8 and DF09 models have the highest degree of colocalization with LbL NPs. OVCAR8 cells display a high degree of colocalization with HA-NPs in both Nude and NSG mouse strains. PDX tumor models established in NSG mice colocalize with PLD-LbL NPs only. Table lists Pearson’s R correlation coefficient (mean ± standard error) for each tumor model


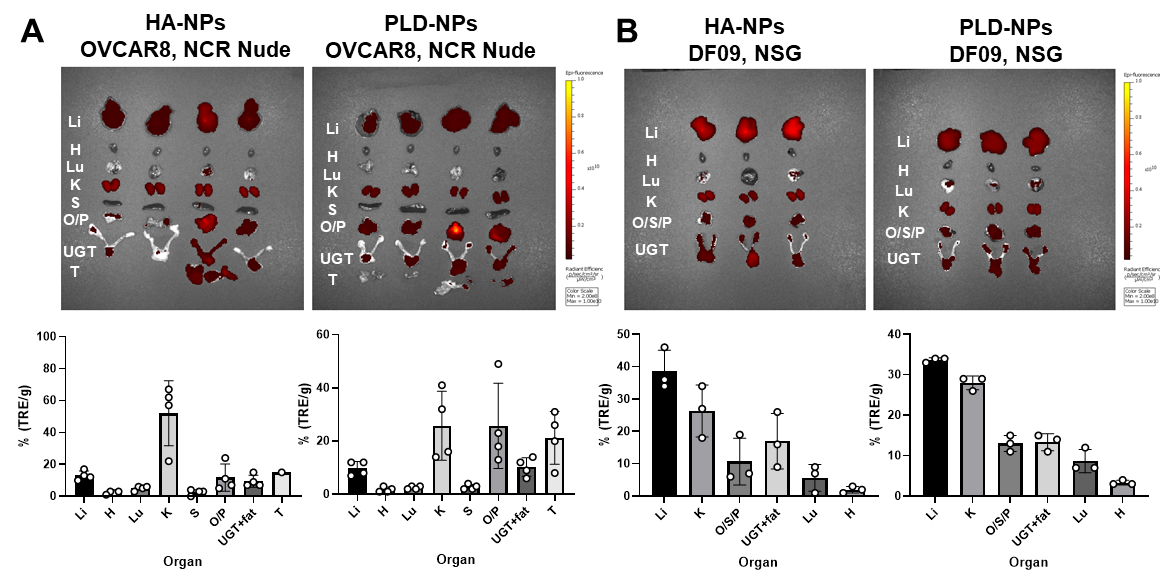


**Figure S3. IVIS images show relative levels of NP accumulation in the heart, lungs, liver spleen, kidney and tumor tissue.** LbL NP signal is normalized by tissue weight. For both (A) OVCAR8-Nude and (B) DF09-NSG models dosed with either HA or PLD-coated NPs, percent total radiant efficiency per gram (% TRE/g) signal was highest in the tumors covering the omentum/upper genital tract, liver and kidney of most models. % TRE/g signal was lowest in the heart, spleen, and lungs. Li = liver, H = heart, Lu = lung, K = kidney, S = spleen, O = omentum, P = pancreas, UGT = upper genital tract, T = tumor


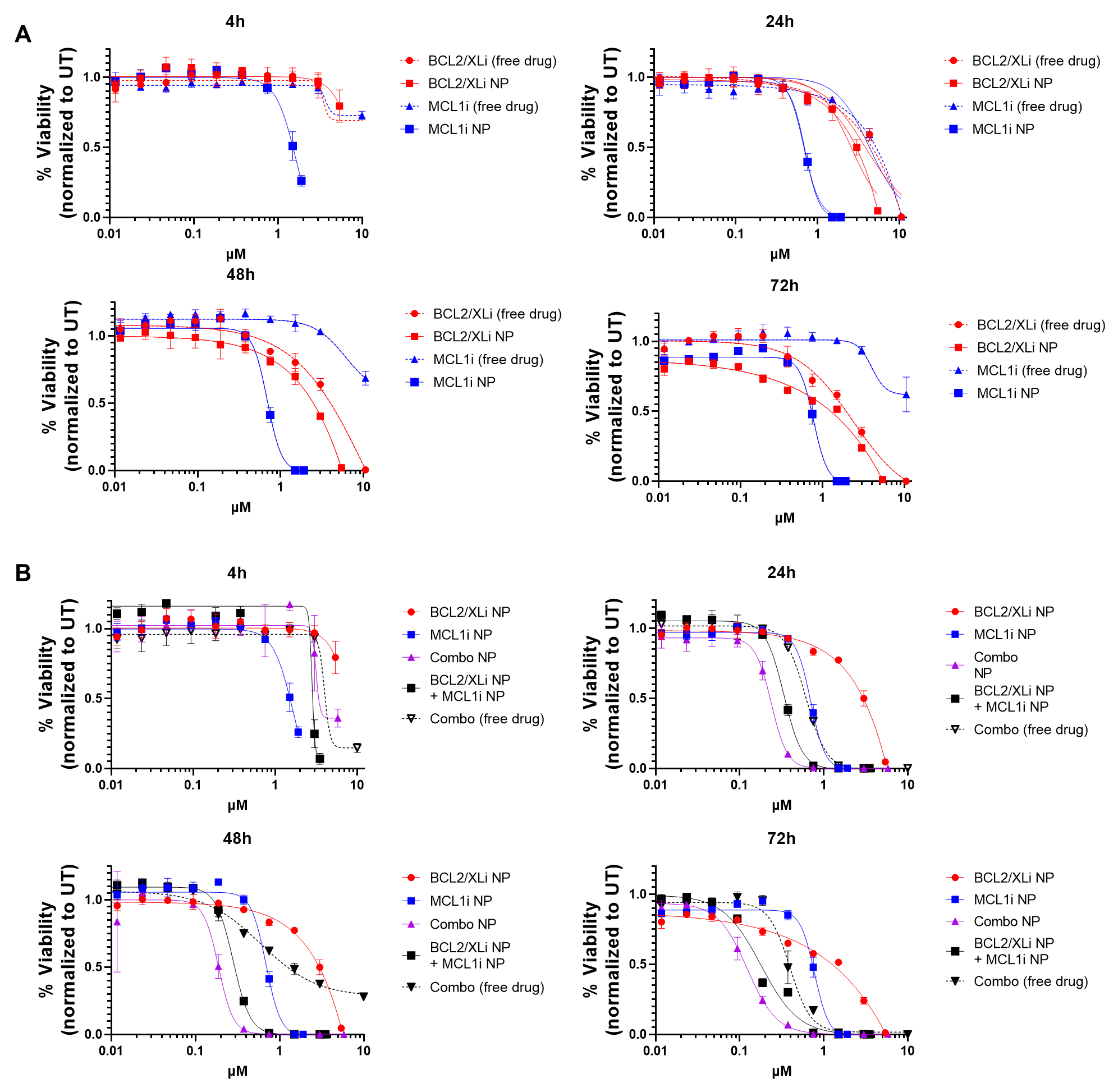


**Figure S4. OVCAR8 cells are treated with drug-loaded HA-LbL NPs and free drug for 4, 24, 48, and 72 hour incubation periods.** (A) For all time points, NP-mediated delivery improved IC50 over the free drug treatment (B) For 24, 48, and 72 hour incubation periods, Combo NP treatment has the highest efficacy—higher than co-treatment with single-drug NPs. Both combination NP treatments and, in some cases, single-drug MCL1i NP treatment had higher efficacy than combination free drug treatment.


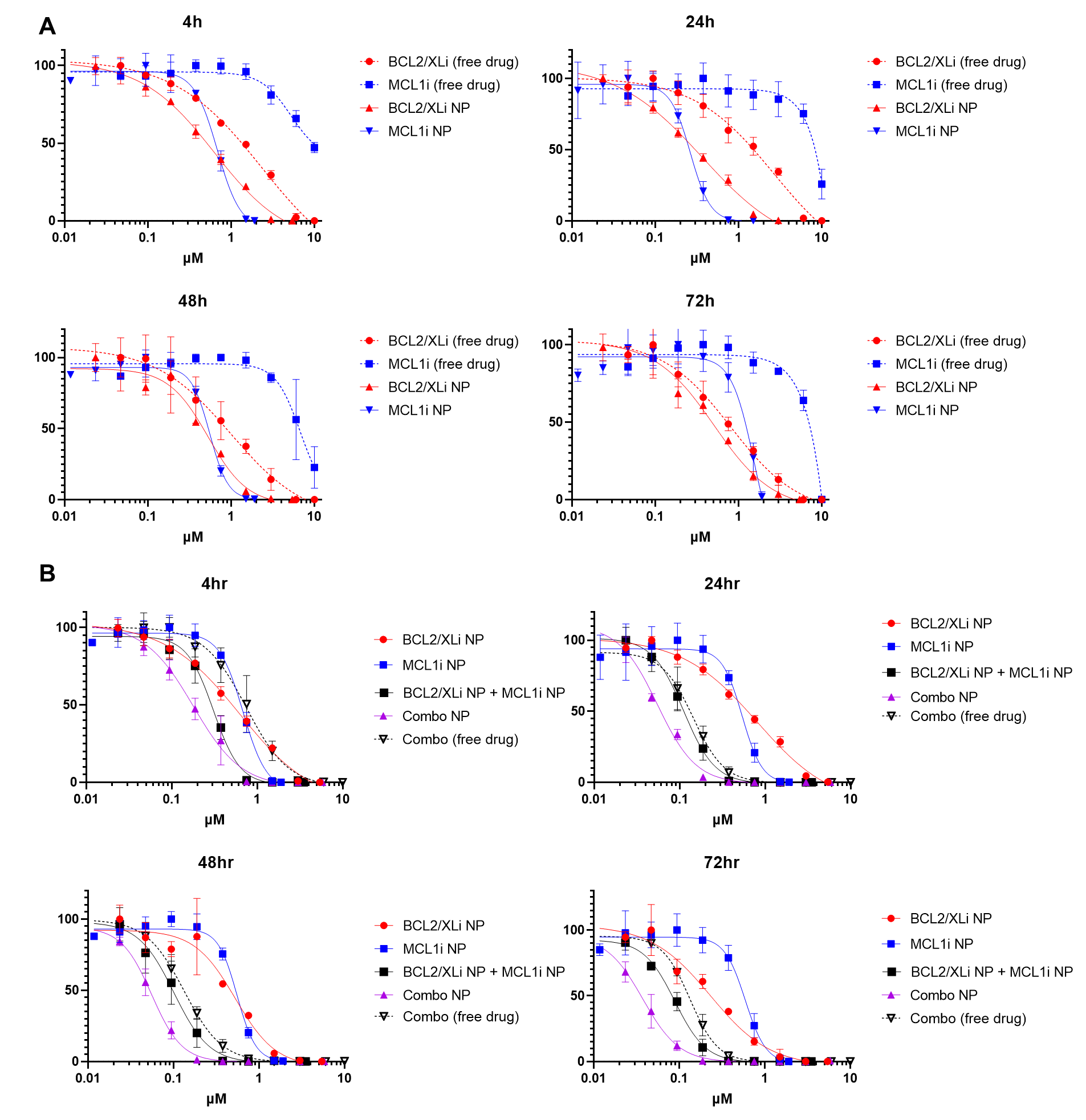


**Figure S5. DF09 cells are treated with drug-loaded HA-LbL NPs and free drug for 4, 24, 48, and 72 hour incubation periods.** (A) For all time points, NP-mediated delivery improved IC50 over the free drug treatment (B) For 24, 48, and 72 hour incubation periods, Combo NP treatment has the highest efficacy—higher than co-treatment with single-drug NPs (BCL2/XLi NP + MCL1i NP), or single-drug NP treatment (BCL2/XLi NP, MCL1i NP).


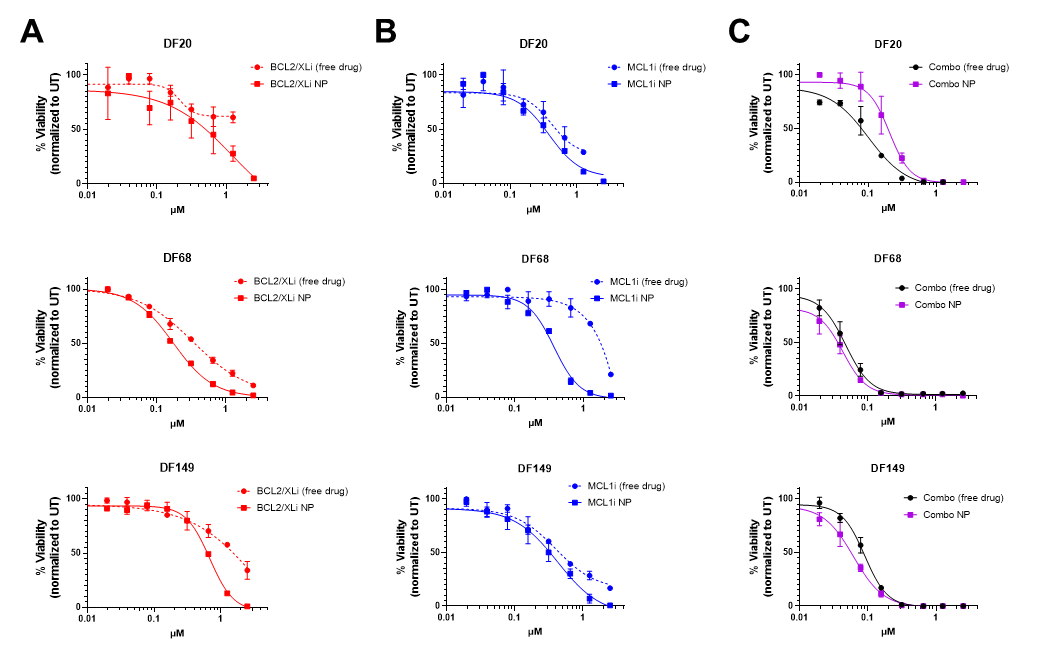


**Figure S6. LbL NP encapsulation and delivery improves therapeutic efficacy of BCL2/XLi, MCL1i, and combination drug treatment for DF68 and DF149.** (A) BCL2/XLi NP has improved IC50 over free drug BCL2/XLi for DF68 and DF149. (B) MCL1i NP has improved IC50 over free drug MCL1i for DF68 and DF149. (C) Combo NP has improved IC50 over free drug combination treatment for DF68 and DF149.


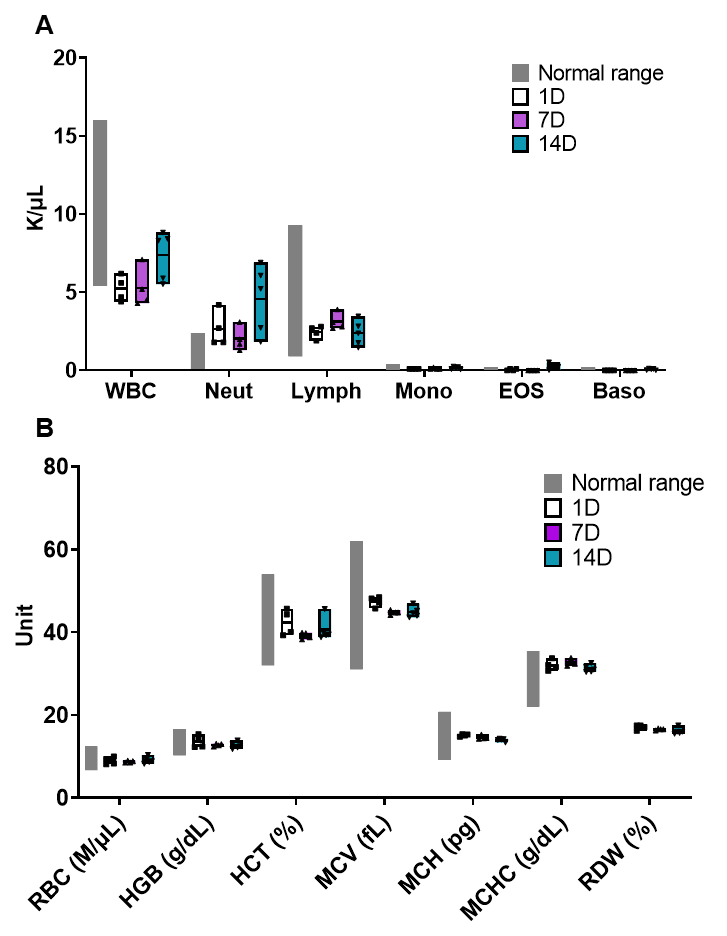


**Figure S7. Blood counts are within normal ranges for 1D, 7D and 14D Nude mice under daily dosing with Combo-NP treatment** (A) Leukocyte levels are within normal ranges (B) Red blood cell levels and morphology are normal


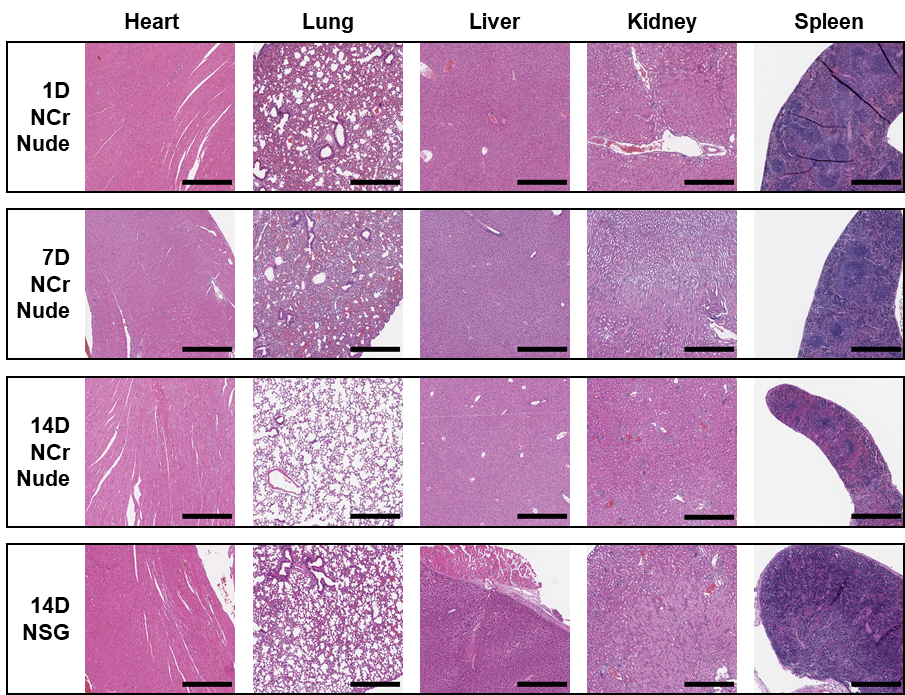


**Figure S8. Histology images show no observable toxicity to heart, lung, liver, spleen and kidney tissue of NCr Nude mice exposed to the Combo-NP.** Tissues were excised from mice bearing OVCAR8-tumors, stained with hematoxylin and eosin, and reviewed by an expert pathologist. NSG mice do not tolerate the Combo-NP drug as well as the NCr Nude mice. NSG mouse liver tissue sections reveal significant inflammation, fibrosis and collagen formation, along peritonitis

**
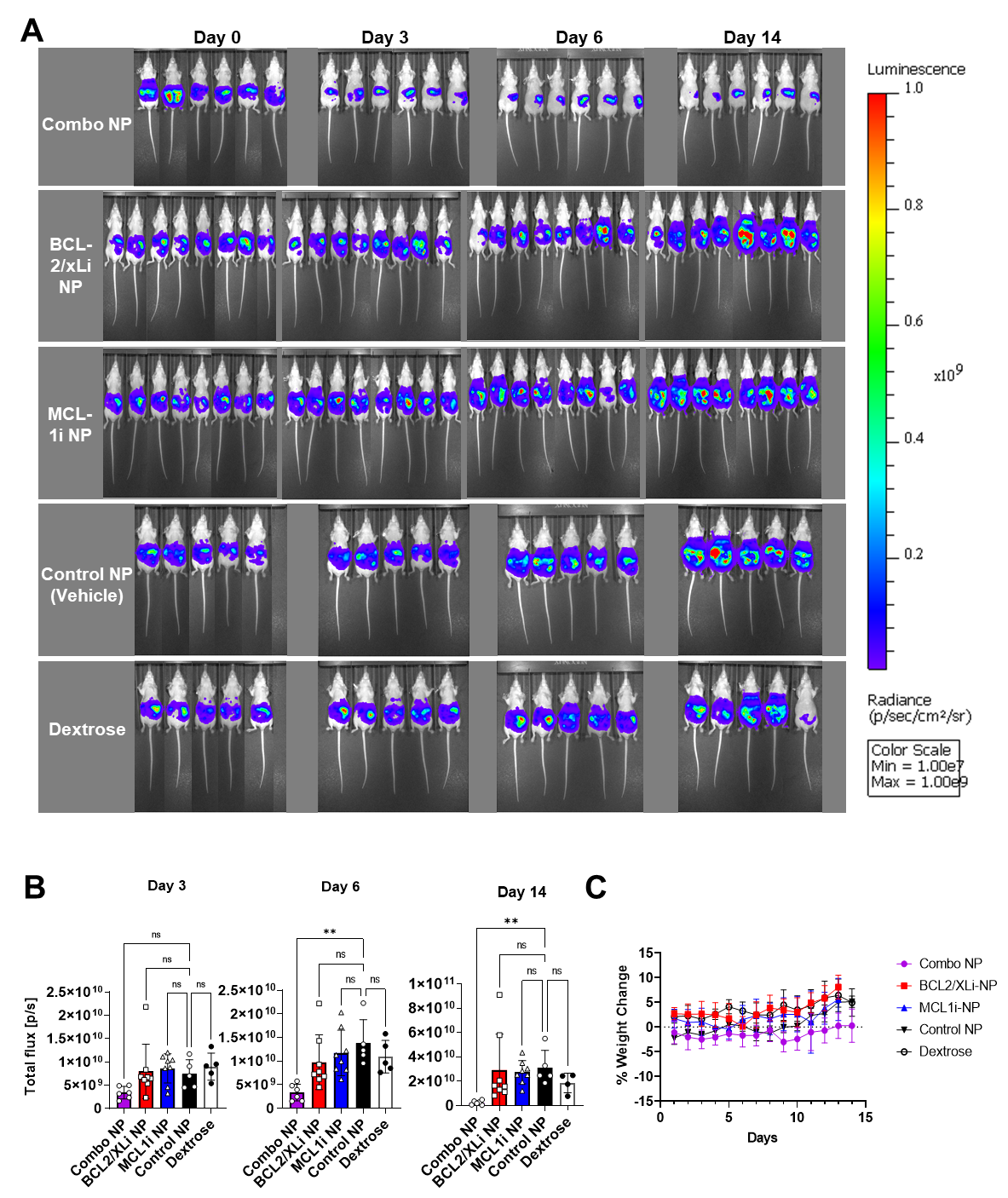
**

**Figure S9. Treatment with Combo-NP significantly reduces tumor burden. Treatment with BCL2/XLi NP or MCL1i NP does not significantly reduce tumor burden.** Tumor burden (BLI signal) was measured throughout treatment course using IVIS. (A) Tumor burden decreases with treatment of Combo NP. (B) Tumor burden grows despite treatment with BCL2/XLi NP or MCL1i NP. (C)Tumor BLI signal of the Combo-NP group compared to both control groups (Control-NP, Dextrose) is significant as early as 6 days after treatment initiation (Combo- vs. Control-NP, P = 0.048; Combo-NP vs. Dextrose, P = 0.007). (D) All treatments are well tolerated. Mice experience no significant change in body weight.


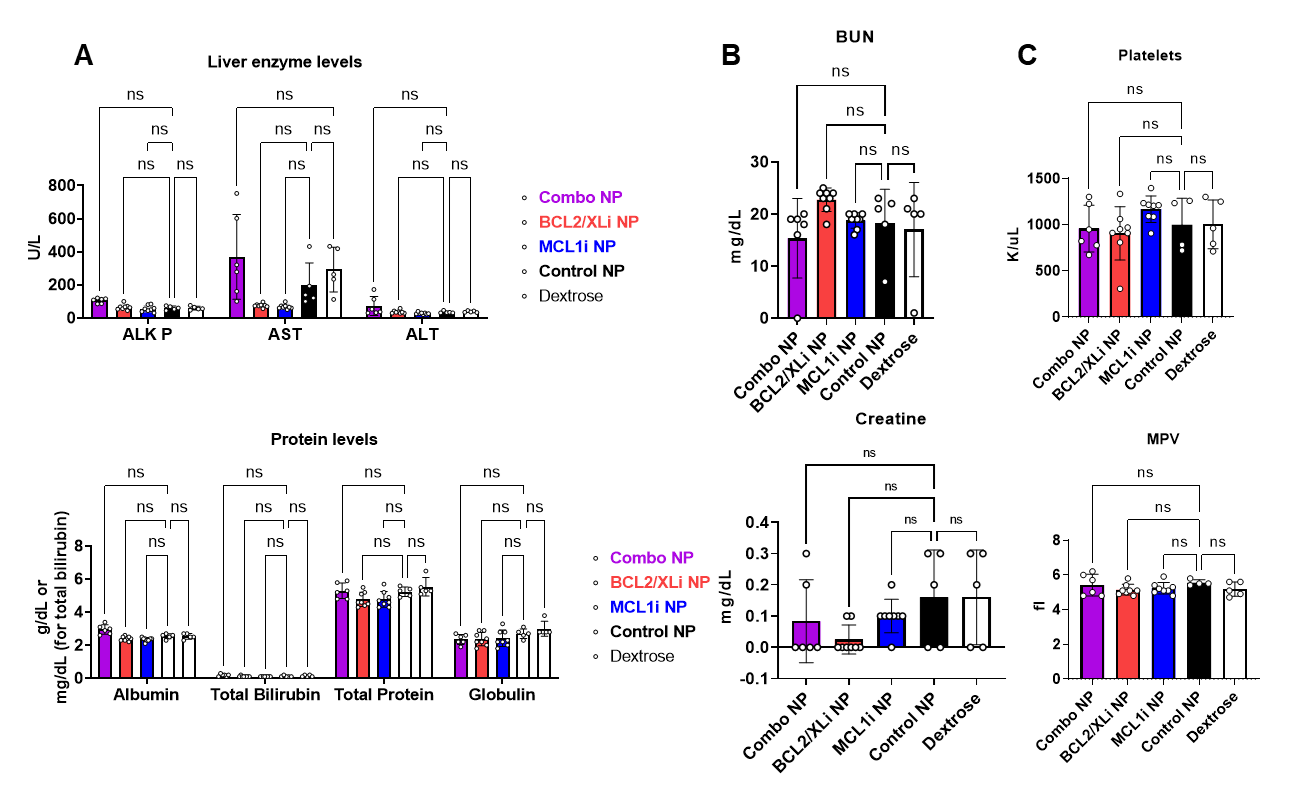


**Figure S10. Liver and kidney function are normal for all treatment groups. Platelet levels and platelet morphology were normal for all groups.** (A) Levels of liver enzymes and levels of albumin, bilirubin, and total protein (indicating liver function) are normal. (B) All treatment groups had normal kidney function, as indicated by BUN and creatine levels. (C) Platelet levels and platelet morphology were normal for all groups.


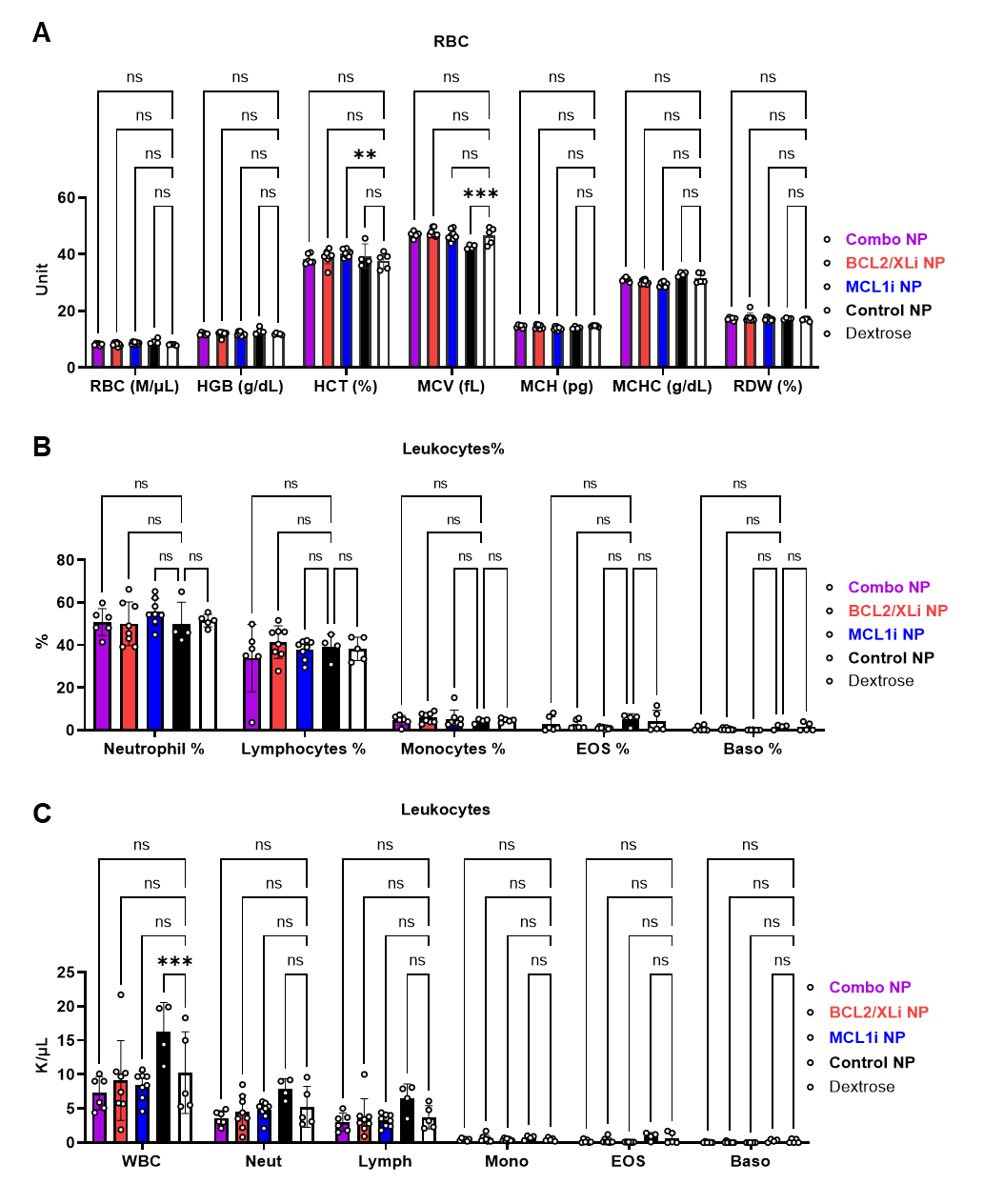


**Figure S11. Blood counts are normal for all treatment groups.** No significant difference in (A) red blood cell health (B&C) leukocyte health were detected among treatment groups.
